# The Unstable Relationship Between Drought Status and Leaf Water Content Complicates the Remote Sensing of Tree Drought Stress

**DOI:** 10.1111/gcb.70188

**Published:** 2025-04-18

**Authors:** Indra Boving, Jean Allen, Philip G. Brodrick, K. Dana Chadwick, Anna Trugman, Leander D.L. Anderegg

**Affiliations:** ^1^ Department of Ecology, Evolution, and Marine Biology University of California Santa Barbara Santa Barbara California USA; ^2^ Department of Geography University of California Santa Barbara Santa Barbara California USA; ^3^ Jet Propulsion Laboratory California Institute of Technology Pasadena California USA

**Keywords:** equivalent water thickness, leaf water content, leaf water content per area, SBG high‐frequency time series, SHIFT, VSWIR

## Abstract

Remote sensing holds promise for ecosystem‐level monitoring of plant drought stress but is limited by uncertain linkages between physiological stress and remotely sensed metrics of water content. Here, we investigate the stability of relationships between water potential (Ψ) and water content (measured in situ and via repeat airborne VSWIR imaging) over diel, seasonal, and spatial variation in two xeric oak tree species. We also compare these field‐based relationships with ones established in laboratory settings that might be used as calibration. Due to confounding physiological processes related to growth, both in situ and remotely sensed metrics lacked consistent relationships with stress when measured across space or through time. Relationships between water content and physiological drought stress measured over the growing season were stronger and more closely related to established laboratory‐based drydown methods than those measured across space (i.e., between wet trees and dry trees). These results provide insight into the utility of “space for time” approaches in remote sensing and demonstrate both important limitations and the potential power of high temporal resolution remote sensing for detecting drought stress.

## Graphical abstract

Remote sensing can monitor plant drought stress across ecosystems but is limited by uncertain links between physiological stress and measurements of water content. We examined how water potential and water content, measured in the field, laboratory, and via airborne imaging, vary over time and space in two oak species. Night‐to‐day field measurements better matched laboratory‐based drydowns than those taken across different locations or between spring and fall. Growth‐related changes in leaf and canopy‐level characteristics weakened these relationships, highlighting challenges in using remote sensing to compare drought stress across locations. Despite limitations, high‐frequency remote sensing shows promise for detecting the development of drought stress through time once leaf growth has ceased.

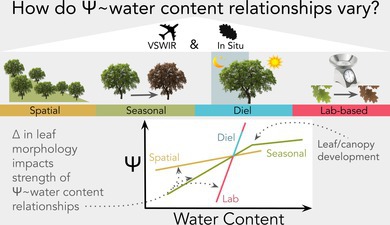

## Translated Abstract

La teledetección tiene un gran potencial para el monitoreo del estrés hídrico en las plantas a nivel de ecosistema, pero su aplicación se ve limitada por la incertidumbre en los vínculos entre el estrés fisiológico y las métricas de contenido de agua obtenidas de forma remota. En este estudio, investigamos la estabilidad de las relaciones entre el potencial hídrico (Ψ) y el contenido de agua (medido in situ y mediante imágenes aéreas repetidas en el espectro visible e infrarrojo cercano, VSWIR) a lo largo de variaciones diarias, estacionales y espaciales en dos especies de robles xerofíticos. También comparamos estas relaciones observadas en campo con las establecidas en condiciones de laboratorio, las cuales podrían utilizarse como calibración. Debido a procesos fisiológicos relacionados con el crecimiento, tanto las métricas in situ como las obtenidas por teledetección carecieron de relaciones consistentes con el estrés cuando se midieron en distintas ubicaciones o a lo largo del tiempo. Las relaciones entre el contenido de agua y el estrés hídrico fisiológico, medido durante la temporada de crecimiento, fueron más sólidas y estuvieron más estrechamente relacionadas con los métodos de deshidratación en laboratorio que con aquellas medidas en distintos sitios (es decir, entre árboles húmedos y secos). Estos resultados proporcionan información sobre la utilidad de los enfoques de “espacio por tiempo” en teledetección y demuestran tanto las limitaciones clave como el gran potencial de la teledetección de alta resolución temporal para la detección del estrés por sequía.

## 1 Introduction

As climate change increases tree mortality globally, measuring tree water status before stress‐related thresholds are reached is key for proactive forest management (Homolová et al. 2013; Junttila et al. 2022; Konings et al. 2019). Remote sensing offers a promising toolkit for doing this, as it can potentially measure drought vulnerability across large spatial scales. Data collected by visible to shortwave infrared (VSWIR) imaging spectrometers have demonstrated the capacity to survey plant biochemistry, productivity, and health at high spatial resolutions (Kokaly et al. 2009; Thompson et al. 2017; Ustin et al. 2004). Yet few remote sensing techniques have been linked directly to tree stress that occurs prior to visible physiological damage (Anderegg et al. 2019; Konings et al. 2021). As a result, we have few leading indicators (i.e., warning signs) of future mortality, which currently rely on observations of trailing indicators (i.e., signals caused by drought damage) (Buitrago et al. 2016; Jones 2004; Liu et al. 2023; Scherrer et al. 2011).

A key uncertainty is how physiological plant stress is linked to various metrics of plant water content captured by remote sensing techniques such as airborne imaging spectroscopy, and whether these relationships are stable over time, across a landscape, or when compared to laboratory settings. Plant water stress is controlled by plant water potential (Ψ), a measure of the potential energy needed to move water through a tree. Ψ has been linked to critical thresholds of tree mortality and is considered the “gold standard” for evaluating tree water status (Hammond et al. 2019; Li et al. 2022; McDowell 2011). Currently, measuring Ψ is time consuming, requires luck in timing measurements around drought events, and is difficult to extrapolate to the landscape level (Anderegg et al. 2019; Binks et al. 2024; Novick et al. 2022). While remote sensing Ψ could revolutionize our understanding of water stress by providing near‐continuous spatial and temporal coverage, adjustments in pressure‐based Ψ are only visually perceptible through downstream effects on plant morphology and structure that occur *after* drought impacts plant performance (Jones 2004). Leaf tissue water content (i.e., the quantity of water in leaves, LW), however, has strong absorptance features across the electromagnetic spectrum and varies substantially with Ψ (Hunt and Rock 1989; Tyree and Hammel 1972). As such, LW is likely a better candidate for remotely sensing drought stress before visible change to the canopy occurs (Konings et al. 2019, 2021).

However, leaf water itself is a complicated quantity. Leaf tissue water content (LW) can be quantified on either a mass basis (LW_mass_, per unit leaf dry mass) or an area basis (LW_area_, per unit wet leaf area) (Table 1). LW_mass_ has been studied extensively by plant ecophysiologists in the form of relative water content (RWC, relative to saturated water content [SWC]), which may be a useful metric for assessing physiological drought damage thresholds (Martinez‐Vilalta et al. 2019; Sapes and Sala 2021) and has been estimated using remotely sensed proxies (Hunt and Rock 1989; Rao et al. 2019). In the laboratory, physiological attributes such as leaf turgor loss point (wilting point) and capacitance (how much water content changes when adjustments in Ψ occur) are often estimated from repeated measurements of Ψ and RWC on an individual leaf during benchtop drydowns (pressure–volume [PV] curves) (Kubiske and Abrams 1990; Tyree and Hammel 1972). However, measuring RWC requires knowledge of SWC, which is challenging to determine due to uncertainty over how to define “maximum” water content (e.g., as a seasonal maximum or following rehydration) (Yang et al. 2016). Thus, absolute LW_mass_ is likely a more actionable mass‐based metric than RWC for upscaling from empirical studies to remote sensing observations. Additionally, LW_mass_ is a focus of fuels and fire‐risk research (where it is termed live fuel moisture [LFM]) due to empirical links with flammability (Boving et al. 2023; Dennison and Moritz 2009) and has been successfully remotely sensed in this context (Roberts et al. 2006; Ustin et al. 1998).

**TABLE 1:** Definitions of in situ and remotely sensed metrics of plant water status and water content.

Metric	Units	Definition	Relevance
Ψ	MPa	Leaf water potential	Pressure‐based measure of plant water status and stress (i.e., tension in the water column)
LW_mass_	g/g	Leaf water content per leaf dry mass, also termed “live fuel moisture”	Physical measure of the amount of water in leaves on a mass basis; used to determine fire‐risk and physiological characteristics (e.g., plant turgor and capacitance)
LW_area_	g/cm^2^	Leaf water content per leaf area	Physical measure of the amount of water in leaves on a leaf area basis
CW_area_	kg/m^2^	Canopy water content per canopy ground area	Physical measure of the amount of water in a tree canopy (LW_area_ scaled to canopy using leaf area index)
CWC	kg/m^2^	Canopy water content	Remotely sensed indicator of the amount of water in a plant canopy on a ground area basis

While many physiological quantities are considered on a mass basis, there are strong reasons from a remote sensing perspective to consider quantities of leaf water per unit leaf area. LW_area_ (the product of LW_mass_ and leaf mass per area [LMA]) may be an important measure of physiological status, but is less often reported due to better established relationships between mass‐based metrics and physiological processes (Lloyd et al. 2013; Osnas et al. 2013; Poorter et al. 2014). However, LW_area_ is likely more readily upscalable to remotely sensed metrics of plant water, which also measure water on an area basis (i.e., per ground or pixel area). Ground measurements of leaf area per unit ground area (leaf area index [LAI]) (Berner and Law 2015; Ryu et al. 2012) and satellite LAI products (Alton 2018; Smettem et al. 2013) are quite common, whereas measurements of leaf mass per ground area are relatively scarce. Thus, translating area‐based physiological quantities into remotely measurable proxies (i.e., multiplying by LAI) is likely far easier than upscaling mass‐based quantities. In theory, whole‐tree canopy water content (CWC) per unit ground area (CW_area_) is the product of LW_area_ and LAI. Because LW_mass_ is a strong function of Ψ in the laboratory, if LMA and LAI are constant, then variation in CW_area_ should be strongly related to Ψ and theoretically measurable by various remote sensing techniques, providing a mechanistic approach for remotely monitoring plant water potential.

Indeed, motivated in part by this theoretical link between canopy water and plant water potential, VSWIR hyperspectral‐derived canopy water content (CWC) has demonstrated promise for remotely monitoring ecosystem drought stress. CWC is a measurement of the amount of water in a tree canopy's leaves per unit ground area (Brodrick et al. 2019; S. Ustin et al. 2012) and is derived from VSWIR imaging spectroscopy by measuring the magnitude of liquid water absorption features in the optical path, known in the literature as equivalent water thickness (EWT, Bohn et al. 2020; Gao and Goetz 1990; Gao and Goetzt 1995; Green et al. 2006). While VSWIR imaging spectroscopy is generally most sensitive to the top of the vegetation canopy, for less densely canopied trees (those with LAI < 6, such as the majority of trees in this study), CWC is thought to capture nearly all liquid water in the leafy canopy while largely excluding measures of woody material (Roberts et al. 2004). Changes in CWC over time (delta CWC [dCWC]) have been observed as a precursor of mortality in some tree species, and CWC has been used to track declines in plant moisture in the context of fire management (Brodrick and Asner 2017; Ustin et al. 2012). One potential benefit of CWC and other metrics derived from VSWIR satellite data (e.g., from NASA's EMIT sensor and planned NASA Surface Biology and Geology (SBG) satellite) over classic multispectral or microwave satellite imagers (such as those used to monitor vegetation optical depth [VOD], another measure of canopy water) is the capacity to estimate canopy water at high spatial and temporal resolutions, which could facilitate tracking shorter term shifts in water content (Rao et al. 2019; Schimel et al. 2019). However, CWC changes might be driven by loss of leaf area or increases in leaf dry matter, and not tissue desiccation per se (Martin et al. 2018). Thus, the question remains: Can CWC measure *water status* (Ψ) before stress results in canopy‐scale consequences? If not, it may have few advantages over existing early warning metrics such as multispectral‐derived nonphotosynthetic vegetation fraction (NPV) or other available metrics of plant health such as VOD (Anderegg et al. 2019; Huang and Anderegg 2012; Konings et al. 2019, 2021; Scherrer et al. 2011; Tucker 1979).

Developing ways to infer plant water potential via remotely sensed diagnostics such as CWC or dCWC would help identify drought vulnerability using leading rather than lagging indicators of plant stress and provide insight into drought effects on the carbon cycle, water cycle, and fire risk (Anderegg et al. 2013, 2019; Konings et al. 2021). Such estimates require an empirically grounded understanding of how water content varies with tree water status (e.g., Ψ and mass‐ or area‐based LW metrics). It remains uncertain whether the relationship between physical metrics of plant water and Ψ is sufficiently stable across space and time to predict differences in Ψ using remote sensing, or if relationships established in laboratory settings (e.g., using PV curves) accurately describe relationships in the field. We hypothesize that correlated and confounding processes of leaf and canopy development (changes in LAI and developmental shifts in leaf morphology such as changes to dry matter content and LMA) result in fundamentally different relationships between plant water content and Ψ across space (i.e., between the wettest and driest trees on the landscape) and through time (as a single tree dries down) compared to the laboratory drydown experiments that have been used for decades to characterize Ψ–LW relationships.

To test this hypothesis, we compared repeated in situ measurements of Ψ with LW_mass_, LW_area_, and estimates of CW_area_, as well as with a unique time series of remotely sensed CWC from the Airborne Visible/Infrared Imaging Spectrometer (AVIRIS‐NG) airborne sensor measured during the spring–summer transition in an oak woodland in southern California, United States. Our sampling focused on a common deciduous oak, *Quercus douglasii*, with a smaller analysis on the evergreen *Quercus agrifolia*. We ask the following: (1) Is the relationship between plant water content and Ψ similar when leaf properties are held constant (over the course of a day, analogous to laboratory drydowns or PV curves), versus across space (between wet and dry trees) versus through time (as a tree dries down and drought stress develops)? (2) Are area‐based metrics of plant hydration, which function as ground‐based analogues to remotely sensed products, better predictors of water potential than mass‐based metrics? (3) Can estimates of CWC from imaging spectroscopy capture variation in drought stress over space and time?

## 2 Methods

### 2.1 In Situ and Remotely Sensed Metrics

**FIGURE 1:**
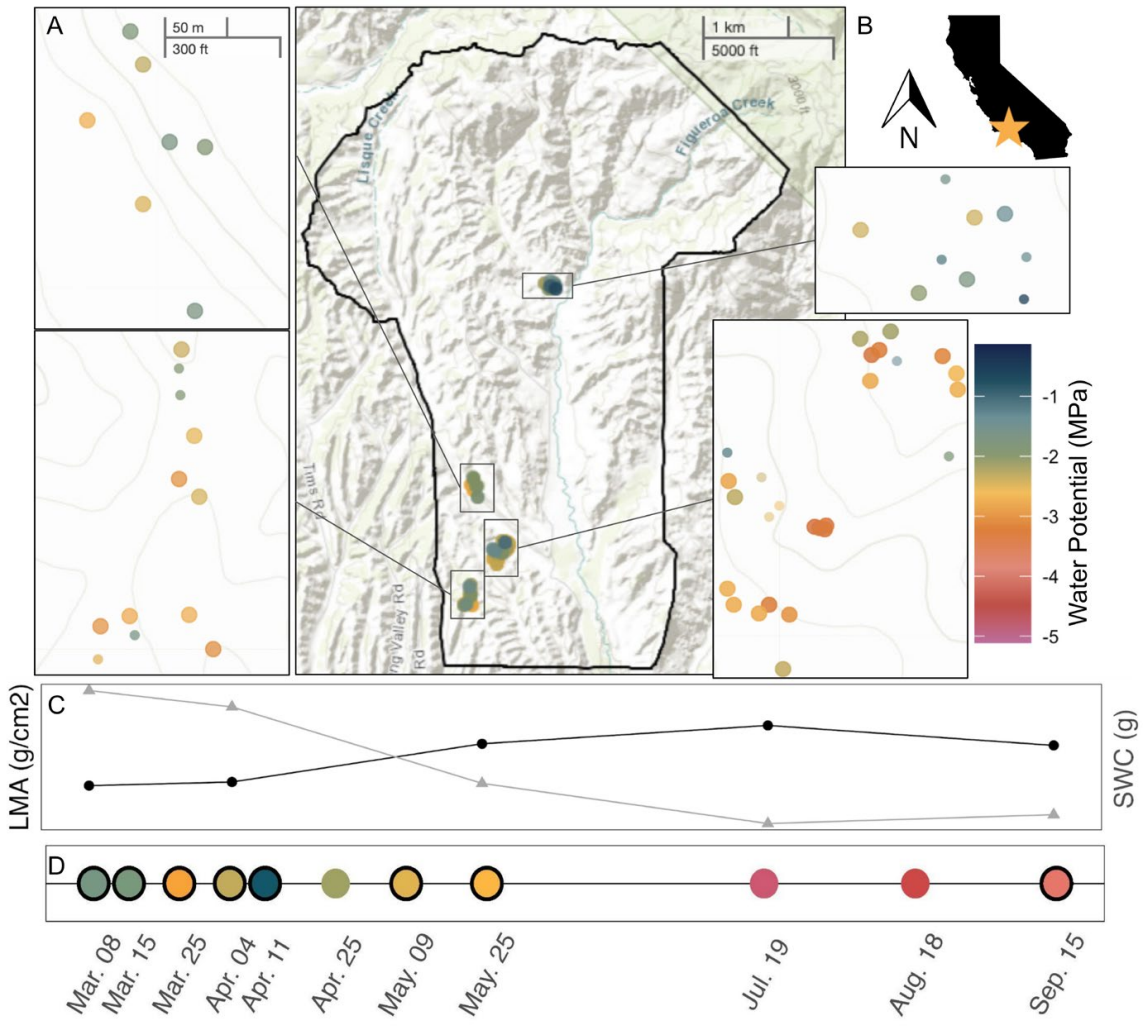
Sampling captured large spatial and temporal variations in water stress and phenology. (A) Map of sampling locations with sites separated by squares, where color scale indicates predawn water potential (Ψ) following leaf expansion (average predawn Ψ for August–September 2022) of individual trees and size indicates species ( *Q. douglasii* = large circles, *n* = 39, *Q. agrifolia* = small circles, *n* = 14). (B) Location of sample site in southern California, United States. (C) Relative change in leaf mass per area (LMA, black circles) and saturated water content (SWC, gray triangles), with mean ± standard deviation over select sampling dates for *Q. douglasii* trees over time. (D) Timeline of sampling, with hyperspectral overflight dates outlined in black and color indicating mean *midday* Ψ across all trees per week; in situ data were collected within 6 days of overflights. Scale bar for all site maps in the upper left panel.

This study took place in a temperate oak savannah in coastal southern California, United States (34.692856° N, −120.040639° W), in conjunction with NASA's SBG High‐Frequency Time Series (SHIFT) campaign. The SHIFT campaign collected repeat VSWIR hyperspectral data using a second‐generation plane‐mountedAVIRIS‐NG sensor at high resolution (5 nm spectral resolution between 380 and 2510 nm at a 5 × 5 meter spatial resolution) over seven time points between March and late May 2022 plus a time point in September (Chadwick et al. 2025). We sampled a total of 39 blue oak trees ( *Q. douglasii* ) and 14 live oak trees ( *Q. agrifolia* ) (Figure 1). Our analysis focused primarily on the winter‐deciduous *Q. douglasii* due to its relative dominance on the landscape and established vulnerability to drought (Anderegg et al. 2023; Dwomoh et al. 2021), while a smaller subset of the less prominent and vulnerable *Q. agrifolia* was sampled for a leaf habit comparison.

The trees are located on a mostly clayey‐skeletal, mixed, active, thermic Ultic Palexerolls, with northernmost individuals located on fine‐loamy, mixed, superactive, thermic Pachic Argixerolls (Soil Survey Staff 2022). Trees are generally in an open stand structure and are surrounded by nonnative grasses that typically undergo green up in late winter to early spring. They cover a range of aspects and slopes, and range in elevation from 325 m at valley bottoms to 450 m at hilltops. The system receives 380 mm of mean annual precipitation and has a Mediterranean climate, with essentially all rainfall occurring between December and March. Our sampling captured a large seasonal drydown, with only 42 mm of precipitation between March and September and no precipitation from May to August. Moreover, the 2021–2022 water year was drier than average (231 mm) and came at the end of a decade of low rainfall that was likely more severe than any drought in the southwestern United States in the last 1200 years (Williams et al. 2022). Consequently, we captured the accumulated drought stress of a decadal drought that persisted until the winter of 2023.

We measured Ψ, LW_mass_, LW_area_, and LMA every 1–3 weeks within 1–6 days of SHIFT overflights from early March to late May 2022, twice more in July and August (which did not coincide with overflights), and then during a final overflight in September (Table S1). Six to nine leaves per tree for predawn Ψ were collected 2–3 h before dawn, and six to nine midday leaves per tree were collected within 1.5 h of solar noon. Branches were clipped from the upper, sun‐exposed canopy and leaves quickly clipped from the branches and were immediately placed in whirlpack bags, which were then placed into ziplock bags that contained a moist paper towel (Rodriguez‐Dominguez et al. 2022). This upper canopy sampling approach was chosen to (a) follow typical water potential collection protocols and (b) measure signals most likely to be captured by remotely‐sensed CWC, which generally captures upper canopy leaves and less so stems or signals within the canopy for trees with higher LAIs (Chadwick et al. 2020; Roberts et al. 2004). Midday leaves were collected in the same manner but were additionally wrapped in foil prior to bagging to minimize transpirative water loss. Four to nine leaves per tree for LMA, LW_mass_, and LW_area_ measurements were collected at the same time and in the same manner as Ψ leaves at both predawn and midday. Equilibrated Ψ was measured using a Scholander pressure chamber (PMS Instruments, Corvallis, OR), 30 min–24 h after sample collection (following Rodriguez‐Dominguez et al. 2022; Ψ was found to remain stable until ~48 h after collection, data not shown). LW_mass_ leaves were weighed for wet mass, then dried at 60°C for 48 h, after which dry weight was measured and LW_mass_ was calculated. While leaf water content (LWC) as it is used in remote sensing studies is typically quantified as grams of water per grams of leaf fresh weight (e.g., leaf dry matter plus water content; Chadwick et al. 2020), here we use LW_mass_ on a unit dry‐weight basis to better compare to physiological and fire‐risk metrics that are on a dry‐mass basis (e.g., LMA and LFM) and to avoid the “moving target” of leaf fresh weight. For leaf area to sapwood area (A_L_:A_S_) sampling, all leaves attached to branchlets from the current growing season were scanned and weighed while fresh, and stem areas were measured using high precision calipers at the basal end of branch samples. Due to sampling constraints that did not allow for immediate scanning of LW_mass_ leaves, LMA and LW_area_ were determined by scanning dried LW_mass_ leaves post hoc, calculating their area in ImageJ (Schneider et al. 2012), and then applying a week and species‐specific conversion factor based on wet area to dry area ratios from proximal A_L_:A_S_ sampling.

LAI was sampled at two time points, early May and September 2022. We measured LAI using a ceptometer (METER, LP‐80) for under‐canopy measurements of solar radiation and a nearby PAR sensor for above‐canopy measurements (Meter Apogie PAR sensor). We averaged together 15–20 measurements per tree to get an estimate of under‐canopy radiation. We calculated LAI using common protocols and a spherical leaf distribution (Campbell 2023). We calculated a ground‐based analogue to remotely sensed CWC, termed CW_area_, by multiplying LW_area_ by LAI (equivalent to multiplying LMA, LW_mass_, and LAI), thereby inferring the amount of water per unit canopy area.

For 11 overflights between March and September 2022 (eight black circled dates in Figure 1 plus two overflights that did not align with in situ sampling on 16 March and 3 May), CWC was retrieved from imaging spectrometry data at 5 m resolution by measuring the magnitude of liquid water absorption features at infrared wavelengths of light (Brodrick et al. 2024). Here, CWC was calculated using the Imaging Spectrometer Optimal Fitting (ISOFIT) Python package (Thompson et al. 2018). ISOFIT calculates CWC by inverting a Beer–Lambert model of light passing through liquid water to solve for water thickness (and accounting the presence of nonwater objects, such as stems) and then fitting that model against AVIRIS‐NG surface reflectance imagery for a range of wavelengths of light where light is absorbed by liquid water (850–1100 nm) to solve for the amount of water in the tree canopy per unit ground area (Bohn et al. 2020; Gao and Goetz 1990; Gao and Goetzt 1995; Green et al. 2006). Due to the relatively low LAI of trees in this study (< 6 m^2^/m^2^), we assume that the impacts of signal penetration depth are relatively low (Roberts et al. 2004).

To assess phenological change, we performed surveys of crown health at six time points during and after leaf expansion, tracking leaf budbreak, expansion, and senescence, and compared these with A_L_:A_S_ and measurements of SWC. To measure SWC, we collected ~80‐cm‐long branches from trees, immediately recut them to ~30 cm segments under water in the field, and stored these samples in a cooler in plastic bags with moistened paper towels. In laboratory, we recut each branch into three branchlets 5–10 cm in length underwater (resulting in three samples per tree) and rehydrated these for 1–3 h with the cut stem in water vials. We measured the saturated wet weight of leaves, dried samples for 24 h at 60°C, and measured their dry weight before calculating SWC as the ratio of water weight to dry weight.

To establish a benchtop‐derived relationship between Ψ and LW_mass_ for the focal species, *Q. douglasii*, we measured PV curves in the laboratory in early April 2022 (during leaf expansion), late May 2022 (after leaf expansion), and then in a more extensive survey in July 2023. We collected branches and rehydrated leaf samples using the same methods as used for SWC. We repeatedly measured leaf mass and water potential as leaves underwent a benchtop drydown (approximately every 0.3 MPa) following established PV curve methods (Tyree and Hammel 1972). Following the drydown, we scanned each leaf and calculated leaf area using imageJ software.

### 2.2 Statistical Analysis

All analyses were conducted in R version 4.3.0 (R Core Team 2023). To determine the impact of phenology, we identified plateaus in leaf expansion by repeated visual surveys of tree phenology and cross‐comparison with changes in LMA, SWC, and A_L_:A_S_ over time (Figures 1C and S1). Leaf expansion was generally complete for the majority of trees by the end of April (i.e., variation across trees in LMA, SWC, and A_L_:A_S_ had condensed), and we considered all samples collected prior to March 1 as “expanding leaves” and samples after that date “after leaf expansion.” To corroborate these dates and to determine the effects of these phenological periods on the relationship between Ψ and LW, we performed variance decompositions using the *vardecomp* function from the variancePartition R package (Version 1.34.0) (Hoffman and Schadt 2016). From this, we could determine how much variation in Ψ was explained by LW_mass_ versus LW_area_ on data from the full date range (March–September 2022) versus after leaf expansion was complete (May–September 2022) (Figures S2 and S3).

To test the diel, seasonal (referred to henceforth as “temporal”), and spatial relationship between LW and Ψ, we built a selection of mixed‐effects models relating Ψ to each water content metric (LW_mass_, CWC, CW_area_, and LW_area_) (Table 2). We also related CWC to CW_area_, to LW_mass_, and to LW_area_ to determine if in situ and remotely sensed metrics of water content were related. To test whether dry‐mass metrics alone could improve predictions of Ψ, we additionally fit models relating Ψ to LMA. We fit all models using the *lmer* function from the lme4 R package (Bates et al. 2014). Each of the mixed‐effects models were designed to disentangle different sources of variation using random effects, with a focus on midday Ψ for seasonal and spatial analyses. For seasonal and diel patterns, we estimated Ψ–water content slopes per canopy for all trees in each time period of interest (e.g., each sampling date for diel and before versus after completed leaf expansion for seasonal), while for spatially driven relationships we analyzed the slope of all trees across a landscape in a given week. We estimated slopes using bootstrapping, randomly sampling trees with replacement over 1000 iterations; bootstrapping was used due to small sample sizes for some weeks and to aid in the visualization of significant relationships in figures via the overlap of 95% confidence intervals with zero. As sample sizes for diel and spatial measurements varied week to week due to field limitations (Table S1), the sample size for random sampling was chosen based on the minimum number of trees collected in a single week over the whole sampling period. We performed similar bootstrapping for the mean PV curve slope and 95% CI, combining curves for all time points (32 curves total) and using simple linear models relating Ψ to either LW_mass_ or LA_area_. We used linear models in the PV curve analysis because the relationship between water potential and water content is generally linear prior to turgor loss in leaf‐level PV curves (Nolan et al. 2022; Tyree and Hammel 1972).

**TABLE 2:** Mixed‐effects models built to determine the effects of different sources of variation on relationships among physiological and water content metrics of drought status: Spatially driven patterns due to variation across a landscape, seasonally driven sources of variation over time, or diel signals due to daily shifts in plant hydration from night to day.

Variation	Model
Space	~ metric + (**metric|week**) + (1|site)
Time	~ metric + **phenology** + (1|week) + (**metric|tree**)
Diel	~ metric + (**metric|tree**) + (metric|week)
PV curve	~ metric + (**metric|tree**)

*Note:* Bolded terms indicate those from which slopes were extracted and visualized in Figures 2, 3, and 5. “Metric” refers to the remotely sensed or in situ metric to be related to drought stress (canopy water content, mass‐based or area‐based water content, or leaf mass per area).

To test whether changes in CWC over time (i.e., the amount of water lost or gained in canopy leaves as the season progressed, dCWC) were predictive of Ψ, we calculated the slope of CWC measured across various timeframes for individual trees: either immediately after leafout (April 29 and three May overflights), for time points before leafout (March 8–April 12), or across the entire spring–summer drydown (all overflights between March and the end of May). We excluded the September sampling due to observed upper canopy leaf browning following a heatwave, which likely impacted CWC retrievals and caused most trees to return CWC values of zero or close to zero. For each timeframe, we calculated the bootstrapped slope of CWC ~ week for individual trees and removed any slope outliers outside the 1.5 IQR. We then ran linear models to determine if the change in CWC for a given tree over a set period of time (dCWC for each timeframe) was predictive of in situ metrics (predawn and midday water potentials, LW_area_, LW_mass_, or LMA). This tested whether a tree's history of water loss could predict future dryness or leaf morphology. As changes to in situ metrics are generally minimal week to week, and to reduce the noise from random variation in the in situ data, we summarized each metric for each tree over portions of the drydown period (early spring from March 8—to April 12, late spring from April 29 to May 29, or for summer from July 19 to September 15).

## 3 Results

### 3.1 Empirical Relationships With Ψ

**FIGURE 2:**
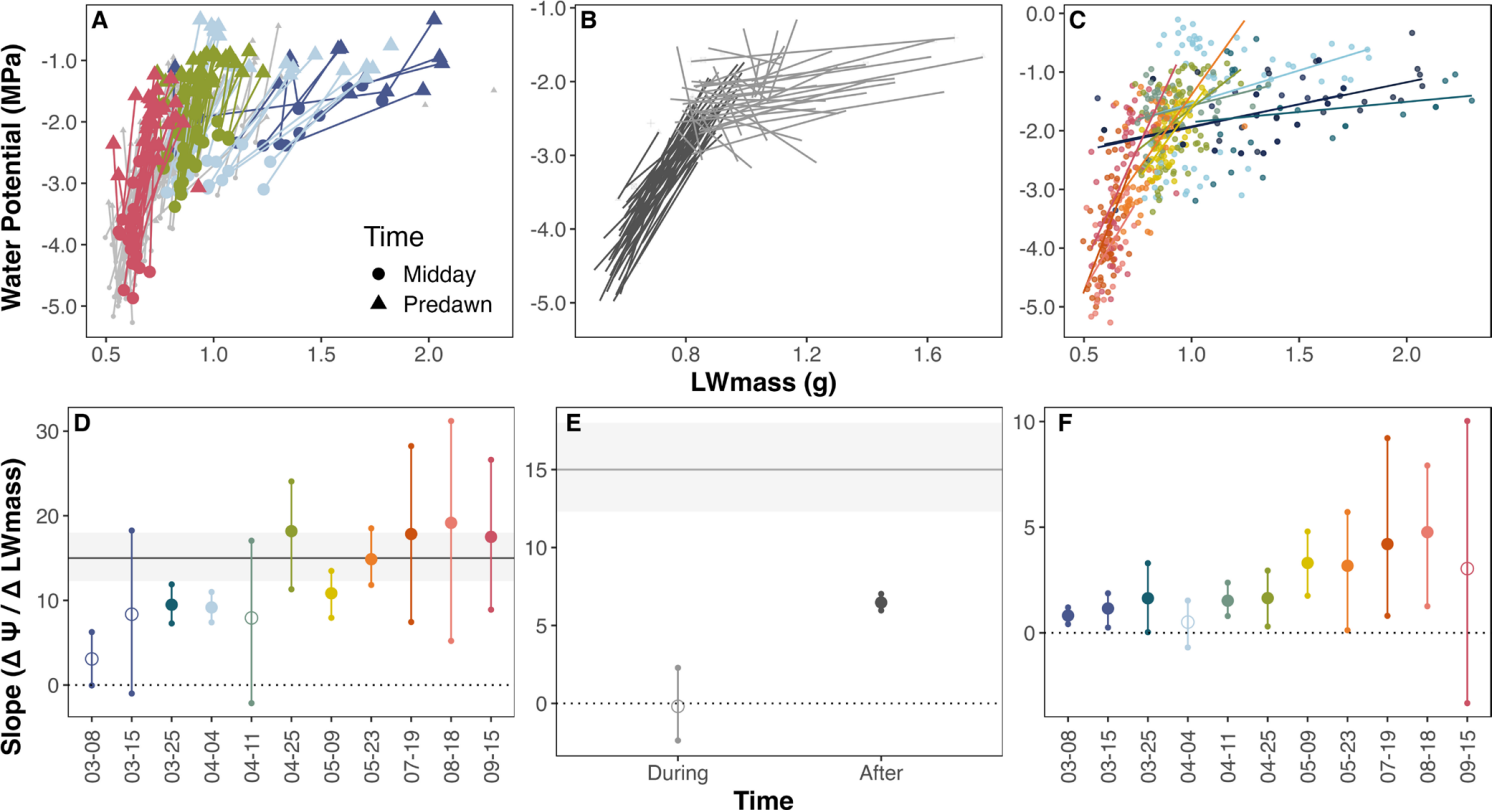
The relationship between leaf water potential (Ψ) and mass‐based water content (LW_mass_) varies across hydration levels, time, and space for *Q. douglasii* . (A) Diel Ψ–LW_mass_ relationship (predawn = triangles, midday = circles) with select weeks highlighted. (B) Relationship between LW_mass_ and Ψ over time for each tree, separated by time periods *during* (gray) or *after* (black) leaf expansion (before or after April 27). (C) LW_mass_ and Ψ relationships among trees for each sampling week (spatially driven variation). (D) Mean slope ± 95% CI across diel tree random effect. (E) Mean slope ± 95% CI across all trees for each time period. (F) Slope ± 95% CI of all trees sampled in a single week. Gray lines and shading on (D–F) indicate the mean slope and bootstrapped 95% CI on PV curves (not visible in F due to scale). Filled points in (D–F) indicate significant relationships (CI does not overlap with zero).

Our sampling captured substantial spatial variation in Ψ (Figure 1A) and a seasonal drydown that resulted in maximum water stress approaching levels observed during a recent drought that induced *Q. douglasii* mortality (2012–2016 when midday water potentials fell below −5 MPa) (Dwomoh et al. 2021; Weitz 2018). During this severe drydown, relationships between Ψ and LW_mass_ were highly influenced by phenological changes in leaf structure and water holding capacity. In *Q. douglasii* , LMA increased and SWC and A_L_:A_S_ decreased during the early season (as leaves were expanding between early March and the end of April, Figures 1B and S1), such that the Ψ–LW_mass_ relationship through time for individual trees showed a strong break point before versus after full leaf expansion. This breakpoint occurred when leaves reached an LW_mass_ of ~0.9 g. After this point, LW_mass_ explained 56% of the variation in water potential (compared to only 19% across the full date range; Figure S2). As a result, the Ψ–LW_mass_ relationship over time was not significant prior to leaf maturation but was strong and consistent after leaf expansion was complete (Figure 2B,E). The evergreen *Q. agrifolia* showed a much weaker effect of phenological period in variance decompositions; yet, like for *Q. douglasii* , the Ψ–LW_mass_ relationship was generally less variable over time in the period after leafout (Figure S3).

For *Q. douglasii* , the Ψ–LW_mass_ relationship across all trees measured in a single sampling period (*spatial analysis*) was often statistically significant in linear models per week (Figure 2C,F, *p*‐value < 0.05), but was especially shallow during leaf expansion and never approached seasonal or diel slopes even after leaves had matured. Moreover, the spatial slope did not stabilize until much later in the year than the average time of leaf maturation (when temporal and diel slopes became more consistent). *Q. agrifolia* showed weaker and more variable spatial trends than *Q. douglasii*. Spatial trends, however, were within the range of after leafout temporal patterns for *Q. agrifolia* (mean spatial trend across weeks: 3.15 ± 3.7 g/g/MPa, after leafout temporal trend: 4.09 ± 3.34 g/g/MPa) (Figure S4C,F).

Leaf expansion also influenced the diel relationship between Ψ and LW_mass_ (predawn to midday with morphology held constant, analogous to classic benchtop drydown curves). In *Q. douglasii*, the daily water potential drop (ΔΨ, proportional to transpiration) increased throughout the season with increasing evaporative demand (ΔΨ in March–April of 1.07 MPa vs. 1.41 MPa June–September, *p* < 0.05). Yet diel changes in LW_mass_ were more drastic earlier in the season, with greater differences between predawn and midday in the spring compared to after leaf expansion (ΔLW_mass_ of 0.25 g March–April vs. 0.10 g June–September, *p* < 0.001). Consequently, the daily Ψ–LW_mass_ relationship was initially quite shallow (large change in LW_mass_ for small changes in Ψ, i.e., high leaf capacitance, where the slope of the Ψ–LW_mass_ relationship = 1/capacitance). The slope became progressively steeper as leaves matured and leaf capacitance decreased (Figure 2A,D). The strong influence of phenology on diel relationships was qualitatively similar in *Q. agrifolia* , despite the presence of substantial prior‐year foliage that did not change during the season.

Slopes from laboratory‐based PV curves conducted on *Q. douglasii* leaves were relatively stable pre‐ and postleaf maturation and across different years (Figure S5). We found that *Q. douglasii* diel Ψ–LW_mass_ relationships overlapped with PV curve relationships for all but the earliest weeks (Figure 2D), while spatial and temporal relationships did not.

**FIGURE 3:**
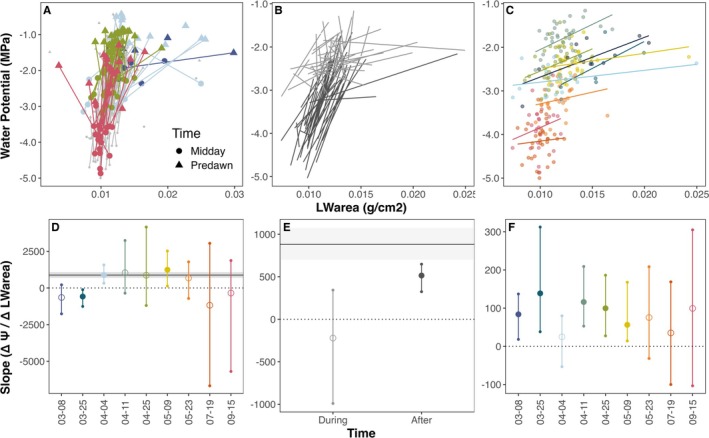
The relationship between water potential (Ψ) and area‐based water content (LW_area_) varies across hydration levels, time, and space for *Q. douglasii* . (A) Diel Ψ–LW_area_ relationship (predawn = triangles, midday = circles) with select weeks highlighted. (B) Relationship between LW_area_ and Ψ for each tree measured over time, separated by time periods *during* (gray) or *after* (black) leaf expansion (before or after April 27). (C) Spatial variation in LW_area_ and Ψ among trees for each sampling week. (D) Mean slope ± 95% CI across diel tree random effect. (E) Mean slope ± 95% CI across all trees for each time period. (F) Mean slope ± 95% CI of trees sampled within a single week. Gray lines and shading on (D–F) indicate the mean slope and bootstrapped 95% CI on PV curves (not visible in F due to scale). Filled points in (D–F) indicate significant relationships (CI does not overlap with zero).

Leaf water content on an area basis (LW_area_) showed considerably less influence of leaf phenology than LW_mass_ (fewer differences in slope across weekly spatial patterns or before vs. after leafout, Figure 3C,F) but also a markedly weaker relationship with Ψ overall (variance contribution: < 5%, Figures S2 and S3). Across spatial sources of variation, Ψ–LW_area_ relationships were only significant for the earliest weeks for *Q. douglasii* (*p* < 0.05) and were never significant for *Q. agrifolia* (Figure S7). LW_area_ also showed little relation to area‐based PV curves for *Q. douglasii* , where both spatially and temporally driven patterns were generally weaker than laboratory‐based relationships, and diel patterns were widely variable and often nonsignificant, even when they did overlap with PV curve slopes (Figure 3D).

**FIGURE 4:**
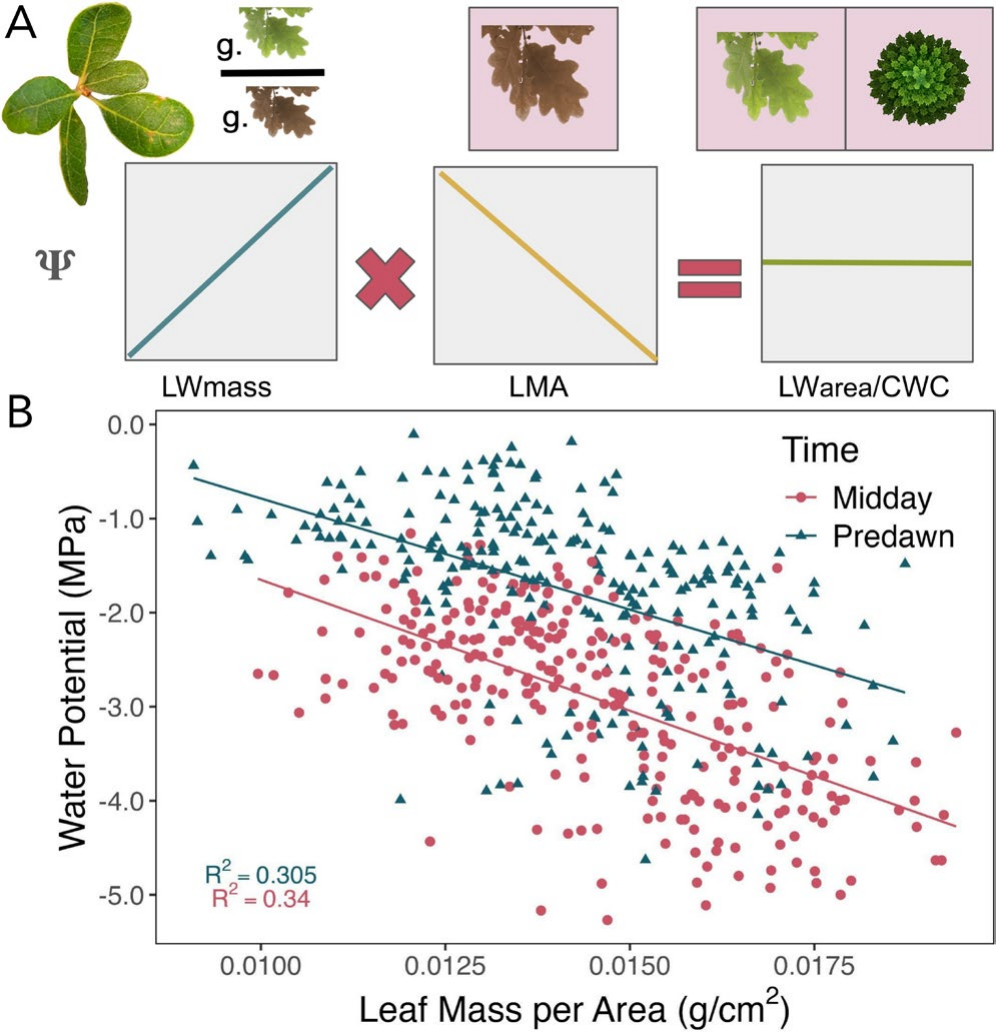
Covariation between water potential (Ψ) and leaf mass per area (LMA) hides relationships between Ψ and area‐based metrics of tissue hydration at both the leaf (LW_area_) and canopy level (CWC). (A) In the deciduous oak *Q. douglasii* (pictured upper left), mass‐based metrics (LW_mass_ and LMA) adjust in tandem during seasonal reductions in water availability, obscuring the relationship between Ψ and area‐based metrics of water content. (B) Relationship between both predawn and midday Ψ and LMA measured in 41 trees over time (*n* = 535).

For *Q. douglasii* , the weaker Ψ–LW_area_ relationship than Ψ–LW_mass_ relationship appears to be driven by negative covariation between LMA and both water availability (predawn Ψ) and maximum water stress (midday Ψ). We found that decreases in water availability and increases in water stress over time corresponded with increases in leaf tissue thickness and density (Figure 4, *p* < 0.001), effectively dampening temporal changes in LW_area_. This level of covariation was not present in *Q. agrifolia*, which showed an overall lower influence of phenology on LW_area_ measurements (Figure S7). In mixed‐effects models comparing the ability of different hydration and morphological metrics to explain variation in Ψ, LMA generally performed better than LW_area_ and CW_area_ (higher marginal R^2^, Table S2), but not as well as LW_mass_ for both species.

### 3.2 Linkages With Remotely Sensed CWC

**FIGURE 5:**
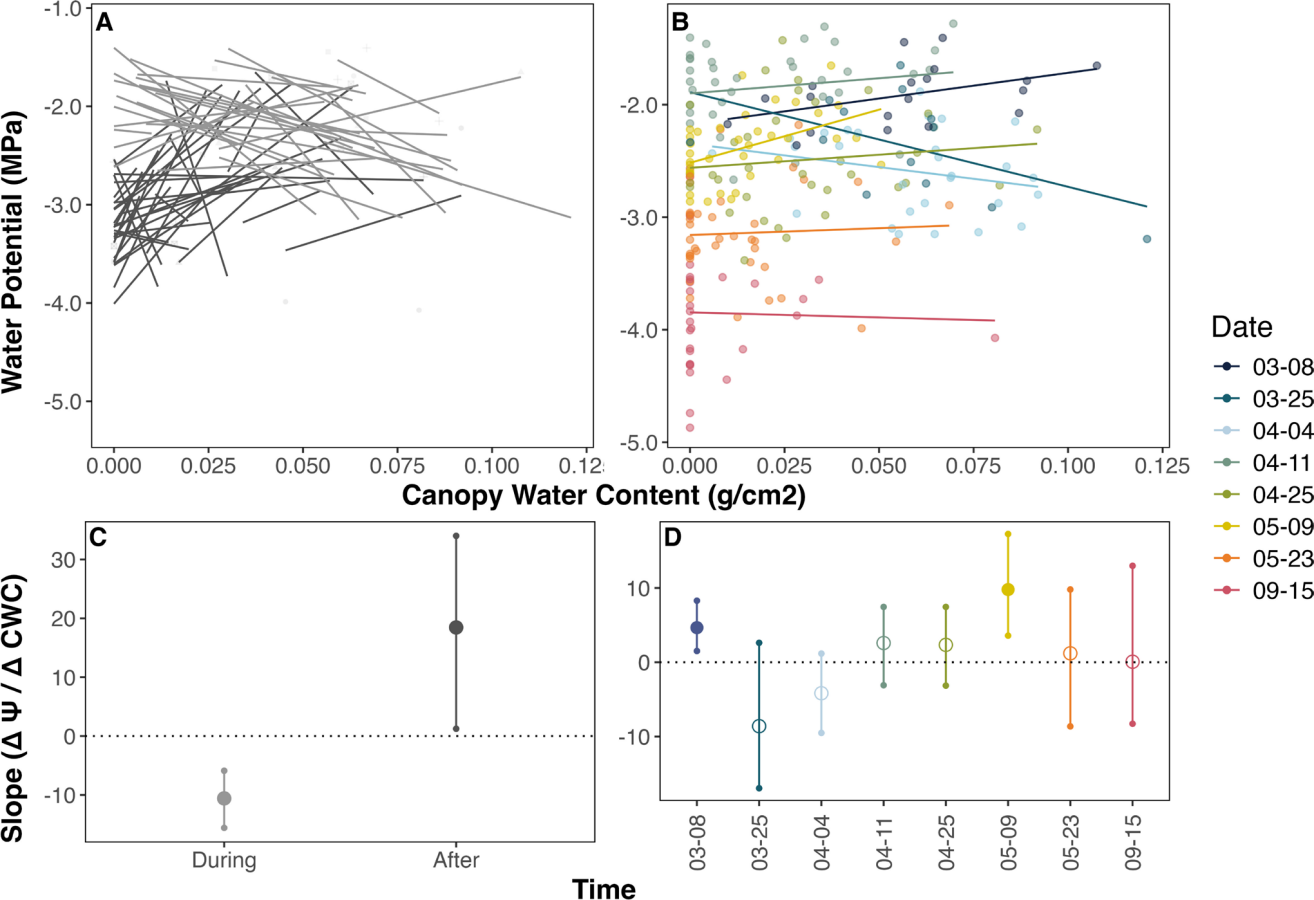
Leaf water potential (Ψ) as predicted by remotely sensed canopy water content (CWC) in *Q. douglasii* . (A) Relationship between CWC and Ψ in individual trees over time before versus after leaf expansion. (B) Spatial variation in CWC and Ψ among trees for each sampling week. (C) Mean slope ± bootstrapped 95% CI across all trees for each time period, separated by *before* and after *leaf* expansion. (D) Slope ± 95% CI of week random effect. Filled points in (C, D) indicate significant relationships (CI does not overlap with zero).

In early season sampling (prior to full leaf expansion), CWC was negatively related to Ψ in *Q. douglasii* , where increased drought stress over time (more negative Ψ) was associated with increased water content (higher values of CWC; Figure 5A,C). Once leaves were fully expanded, Ψ–CWC relationships showed significant and expected trends (more negative Ψ in trees with lower CWC, *p* < 0.05) but were highly variable across individual trees. In the spatial analysis, we found few significant Ψ–CWC relationships across trees measured on a single date (Figure 5B,D). For *Q. agrifolia*, temporal patterns were nonsignificant during either phenological period or across space (Figure S8). When measured across the landscape on a single date, CWC was also unrelated to both metrics of LW for both species (Figures S9–S12). Temporally driven patterns, however, were significant for LW_mass_–CWC in *Q. douglasii* , showing positive and significant relationships for time periods both before and after leafout (Figure S9, *p* < 0.05).

We expected to see strong relationships between CWC and a ground‐based CW_area_, as they are both estimates of whole‐CWC per unit ground area. However, we found that CWC–CW_area_ relationships were not significant over space or time for either species (Figures 7 and S13). CW_area_ was significantly related to *Q. douglasii* Ψ over time (Figure S14), mirroring relationships we found for LW_area_, but was unrelated across space for *Q. douglasii* or for any source of variation for *Q. agrifolia* .

**FIGURE 6:**
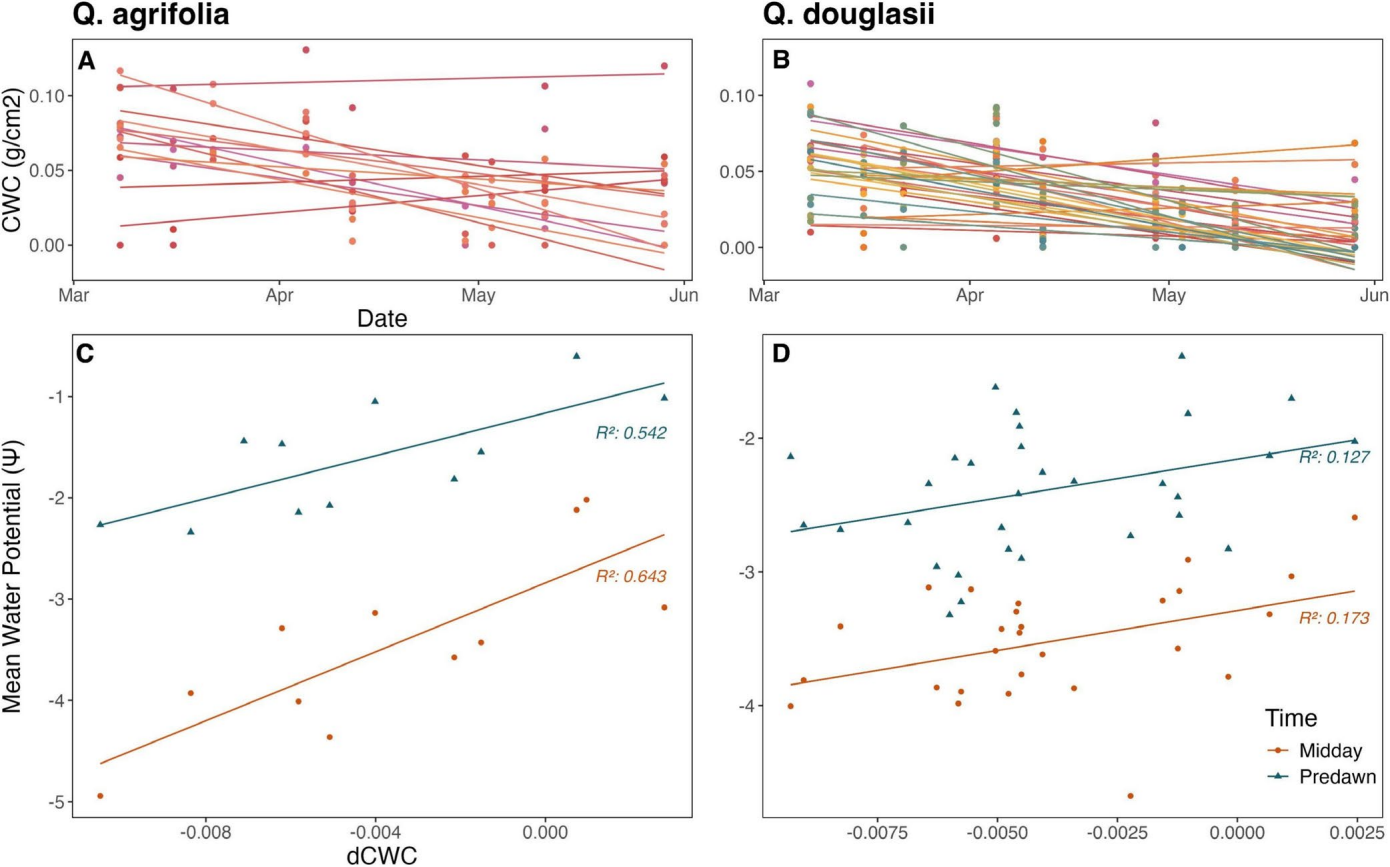
Change in canopy water content over time (dCWC) from March to the end of May (A, B) is related to in situ measured predawn and midday water potential for the evergreen *Q. agrifolia* and *Q. douglasii* averaged over the summer months (C, D), meaning canopy water loss during the spring predicts summer water stress. dCWC (y‐axis in panels C and D) is equal to the slope of lines shown in panels (A, B), while water potential values are averaged across in situ sampling dates from the end of the summer dry period (July 19, August 18, and September 15). R squared shown in the color of their corresponding linear model, colored based on time of day (predawn = blue, midday = red). Relationships visualized in (C and D) are significant (*p* < 0.05).

Change in CWC over time (dCWC) was an overall poor predictor of spatial variation in in situ metrics for *Q. douglasii* , including LMA. However, when in situ metrics were averaged across multiple sampling dates (mean predawn or midday Ψ in early spring, late spring, or summer), spring dCWC calculated (CWC change from March 8 to May 29) was significantly related to summer predawn and midday Ψ (Figure 6). Spring dCWC was also significantly related to early season predawn LW_mass_, late spring and summer midday LW_mass_, and late spring LW_area_ (Figure S15).

For the evergreen *Q. agrifolia* , dCWC was a slightly stronger and more consistent predictor of spatial variation in water potential compared to *Q. douglasii* , but was similarly not strongly related to other metrics (Figure S15). dCWC in *Q. agrifolia* showed consistent patterns with water potential, where observations of greater water loss (more negative dCWC) always corresponded to drier trees (more negative predawn and midday Ψ) (Figure 6). Only dCWC calculated from pre‐leafout overflights (March 8–April 12) was able to predict changes in LW_mass_ and LW_area_, and only for late spring or summer in situ samplings. dCWC was unable to predict LMA for either species or time combinations.

## 4 Discussion

**FIGURE 7:**
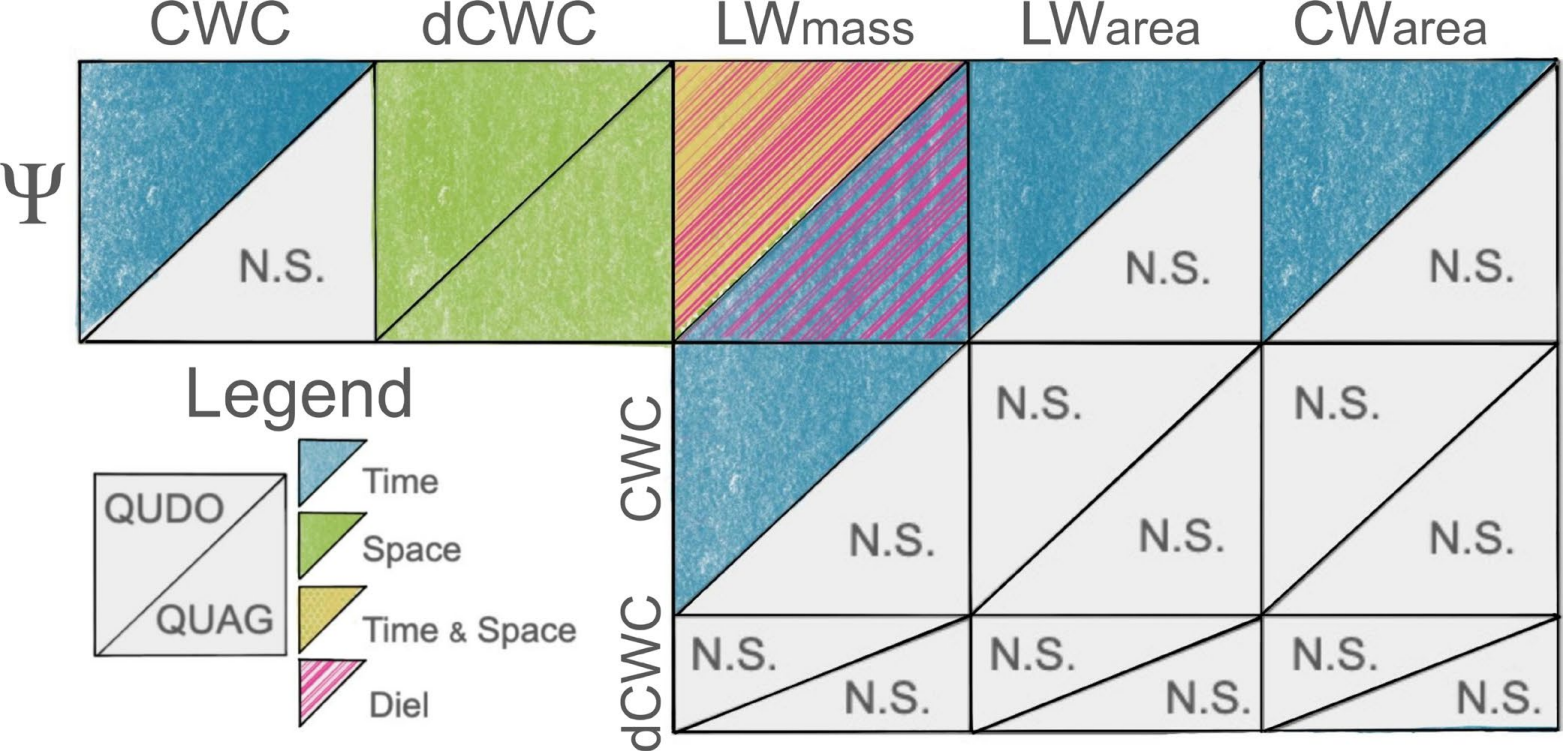
Summarized results showing significant relationships among in situ and remotely sensed metrics of physiological drought stress for *Q. douglasii* and *Q. agrifolia* . Relationships are visualized if late‐season sampling (end of summer, 2022) was generally significant and in the correct direction. Colors indicate the source of variation over which variables have a significant relationship: Over time (blue), across space (green), across space and time (yellow), or driven by diel changes (pink lines). dCWC relationships are considered “spatial” as predicted variables are measured in a single time period. Corner triangles of each box indicate species, with upper right triangles for *Q. douglasii* (QUDO) and lower left for *Q. agrifolia* (QUAG). Uncolored triangles have no significant relationships (labeled N.S.).

Using a novel time series of synchronous in situ and remotely sensed metrics of tree water status and water content, we test key unknowns in remote sensing drought stress: (1) the agreement among spatial and temporal signals and the utility of space for time approaches, (2) the potential for relationships determined in laboratory settings to be used in field‐based analyses, and (3) potential physiological underpinnings of remotely sensed signals. We find that these relationships vary based on how drought stress is measured and what sources of variation are of interest (Figure 7). These findings highlight that the simple relationship between leaf water content and leaf water potential is strongly modified by variation in leaf morphology through space and time. Consequently, remotely sensed metrics of vegetation water are correlated with plant water potential through time for a single tree but not in a straightforward and consistent manner. These results provide broader insight into the potential power and likely limitations of VSWIR imaging spectroscopy‐derived CWC, as well as other types of remote sensing approaches that are sensitive to vegetation water content.

### 4.1 Phenology Is an Essential Driver of Hydration Signals

Our spatial and temporal sampling revealed important phenological signals in both in situ and remotely sensed metrics of drought stress and water content. For *Q. douglasii* , the deciduous oak species that was the focus of our study, we saw a large impact of phenological period on water stress signals, particularly in the phenological period between budbreak and the end of leaf growth when both leaf morphology and canopy LAI were changing. These signals were present to a lesser extent in the evergreen oak species, *Q. agrifolia* , likely due to its fuller canopy and repeated leaf flushes. Processes related to leaf expansion and resulting covariation between Ψ and LMA likely masked signals between drought stress and area‐based metrics of LW. Perhaps because of this, LW_area_ showed more consistent relationships with water potential across time (fewer differences between phenological period in variance decompositions, Figures S2 and S3) but was more weakly related to Ψ overall compared to LW_mass_. These results indicate that remote sensing drought status will be most successful when phenological change has stabilized such that relationships between water content and Ψ are mostly driven by hydration and not variation in leaf dry matter, and where mass‐based metrics might be estimated via LAI and LMA. In our study system, this would be late summer or autumn, which is also when drought stress is most likely to be of concern.

Our finding that LW_area_ was differently related to Ψ than LW_mass_ is compatible with the history of area versus mass‐based metrics, which have long been a source of debate in plant ecology (Osnas et al. 2013; Poorter et al. 2014). While area and mass‐based metrics can offer insight into different physiological processes (e.g., in the leaf economics spectrum, Wright et al. 2004), mass‐based metrics are more common than area‐based metrics (e.g., ~90,000 observations of leaf nitrogen per unit area versus ~165,000 observations per unit mass in the TRY global trait database (Fraser 2020)). More emphasis should be placed on these differences to articulate how and when each normalization is used and how it might impact research findings.

### 4.2 Temporal and Spatial Signals Across In Situ Hydration Metrics

Spatial Ψ–LW relationships were universally different from temporal Ψ–LW relationships for both species, indicating that space for time analyses should be approached with a nuanced understanding of nonstationary ecological processes. For both species and in situ metrics of LW, temporal patterns after leaf expansion were strong and consistent, while spatial relationships were generally weak and varied across sampling periods, stabilizing much later in the year than the average time of leaf maturation. Edaphic and climatic variation across a landscape drives tree‐to‐tree differences in drought status and in morphological responses to prior stress (e.g., increased in leaf dry matter, leaf shedding, necrosis), factors that likely impact the stability of Ψ–LW relationships across space versus through time. Differences in water availability or temperature across space likely confound temporal signals by accelerating or delaying phenology, or by impacting leaf morphology through nutrient pathways. Our trees were dispersed over a diversity of elevations and aspects, which likely exacerbated the effects of microclimate on tree–tree variation in phenology.

While the problem of nonstationarity in ecology is not a new one (Palacio et al. 2008; Rollinson et al. 2021), our work shows that it is especially important to consider in remote sensing studies: barring repeated sampling over time, researchers should aim to identify periods of the least phenological change to use for inferences on temporal variation. In our study system, temporally driven Ψ–LW_mass_ relationships *after* leaf expansion showed the most promise for linking to late‐season spatial patterns (e.g., spatial relationships began to approach temporal relationships), likely due to stabilization of phenological signals across trees on the landscape. For *Q. agrifolia*, despite generally lower sample sizes, we found slightly more consistent temporal and spatially driven relationships for Ψ–LW_mass_, likely due to lower influences of phenology in this evergreen tree.

### 4.3 Laboratory‐Based Methods for Calibrating Field Relationships

Phenological signals also emerged in diel relationships across the season. In *Q. douglasii* leaves, late‐season diel relationships agreed with laboratory‐based patterns. This is a promising sign for applications in remote sensing, as measuring the difference between predawn and midday water potential (e.g., diel transpirative water potential drop) using calibrated relationships in the laboratory can then provide insight into landscape‐level drought stress. Relationships established using laboratory PV curves can likely be used to approximate diel patterns of drought stress following leaf development in campaigns using sensors capable of collecting data during both day and night overflights (e.g., microwave‐based retrievals of VOD). However, we found that PV curves might overestimate capacitance when measured on immature leaves, indicating that attempts to measure diel hydration should be focused on time periods with minimal phenological change and that species‐level relationships should be determined for different time periods.

While useful for determining when diel patterns might be linked to laboratory‐based ones, phenology and other confounding morphological signals swamped any similarities between spatial or longer term temporal field patterns and those measured in the laboratory. Importantly, we found that neither temporally nor spatially driven Ψ–LW_mass_ or Ψ–LW_area_ slopes (or Ψ–CWC, for that matter) reached those of in situ diel slopes or those measured in PV curves, indicating that laboratory relationships are not good approximations for spatial and temporal patterns of drought stress. Many factors likely contribute to this: PV curve slopes are largely a function of individual leaf SWC, elasticity, and capacitance, which vary within canopies, across individuals, and over time. Additionally, water potential gradients at the canopy level likely make PV curves a rough proxy at best for trees that are at disequilibrium (Binks et al. 2024). Determining the drivers of PV curve slope variation at each of these levels and timeframes, as well as the influence of leaf traits on the PV slope, will be important for calibrating larger scale patterns. For passive remote sensing (and any sensor restricted to daytime overflights), field‐based campaigns that explicitly measure either spatial or temporal variation will be necessary to ground truth and calibrate remotely sensed data products seeking to measure drought stress across a landscape. Alternatively, better understanding drivers of variation in PV curve slopes over time, space, within, and across individuals might allow for calibrated relationships to be established using a combination of laboratory data and standardization procedures.

### 4.4 Canopy Water Content Is Variably Related to In Situ Metrics

Our finding that CWC was a generally poor indicator of water potential across both spatial and temporal sources of variation underscores that caution should be used when interpreting remotely sensed CWC. Stable LAI is necessary for pixel‐based sensing to capture accurate change over time in a tree canopy smaller than that pixel, and we found that increases in canopy leaf area during the early growing season and cooccurring covariation between LMA and Ψ confounded hydration‐related signals in CWC over time. There was likely also a strong influence of understory grasses in CWC retrievals: *Q. douglasii* canopies are sparse until leaf expansion is complete, and hyperspectral signals likely captured patterns of vegetation below tree canopies. Indeed, understory green up and senescence occurred during the period when we observed inverted relationships between CWC and drought stress. However, the early spring Ψ–CWC slope was negative on average even in the denser canopied evergreen *Q. agrifolia* (Figure S8), where understory contamination was likely minimal.

Even when drought was well underway—and regardless of cross‐landscape variation in visible changes to canopy structure, such as leaf shedding and browning—we saw no significant spatial patterns in Ψ–CWC relationships for either species. The lack of a Ψ–CWC relationship across trees on the landscape is largely unsurprising, given that trees with vastly different canopy biomass, and therefore canopy water, might have the same Ψ unless canopy biomass is itself correlated with Ψ. A few additional processes likely also weakened this relationship: that differences in canopy development and leaf area across trees swamped the influence of Ψ on CWC, that cross‐tree variation in CWC was too low to be detectable, or that understory contamination was large. These combined make CWC an overall poor indicator of tree stress and must be well understood before CWC can be used to infer the types of changes in drought status that preclude loss of physiological function and mortality. CW_area_, which incorporates differences in canopy area and biomass via LAI, was also largely unrelated to CWC. This speaks to both the difficulty of retrieving CWC and the potentially compounding errors of measured LW_mass_, LMA, and LAI behind CW_area_. Two components of CW_area_, namely LW_area_ and LMA, both had strong relationships with Ψ individually, which likely accounts for our observation that CW_area_ had a stronger relationship with Ψ over time than did CWC (Table S2).

While previous studies have shown that changes in CWC over time (dCWC) can capture variation in drought stress better than static CWC (Bright et al. 2017; Brodrick and Asner 2017), we found mixed evidence in our oak species. Overall positive and often significant relationships for Ψ–dCWC, most consistently in the evergreen *Q. agrifolia* , bolster the case for dCWC as an indicator of water stress when species have canopies that are sufficiently full and stable for reliable repeat measurements of CWC (e.g., in evergreen trees or deciduous trees after leaf maturation). However, Ψ–dCWC must be able to distinguish between predawn or midday Ψ to truly provide insight into water stress, as predawn and midday water potential are indicative of distinct environmental factors (soil water availability vs. atmospheric water demand and stomatal responses), both of which we found to be linked to prior water loss. These need to be distinguished to interpret dCWC in the context of physiological drought response. Nevertheless, the ability to link a tree's history of water loss (e.g., dCWC) with future drought stress (water potential estimates) represents an important step for understanding the spatial distribution of drought stress.

Our results suggest that both static CWC and dCWC are likely complicated metrics to interpret when canopies are developing, when understory vegetation is undergoing drastic changes, and if cross‐tree morphological heterogeneity is large. Overall weak linkages between static CWC and in situ metrics, despite some strong mechanistic and theoretical relationships, indicate that effort should be focused on canopy‐level signals of drought stress and that these might not necessarily be directly coupled with signals at the leaf level. For example, water stress alters tree carbon allocation, leading more water‐limited trees to use conservative leaf allocation strategies and resulting in lower overall leaf area relative to stem area (A_L_:A_S_, or whole‐tree LAI) (Rowland et al. 2023; Trugman et al. 2018; Williams et al. 2022). Tracking these changes using remote sensing may be a more feasible option than tracking Ψ itself. Additionally, by defining axes of trait coordination that occur at the canopy level (e.g., between physiological traits related to drought tolerance and leaf carbon allocation), remote sensing water status might even be possible earlier in the season or in systems with multiple leaf flushes. Our results reinforce the need for repeat sampling of CWC and suggest that capturing variation in drought stress across individual trees will require both controlling for biomass/LAI and extensive parameterization of tree morphology.

### 4.5 Broader Applications in Remote Sensing

Collectively, our results suggest a few critical points about remote sensing plant water stress. First, techniques that have day and night overflights, such as microwave‐based VOD, can likely measure diel changes in water content analogous to classic PV curves because both biomass and morphology are held constant (Konings et al. 2021; Rao et al. 2019; Wood et al. 2024). However, understanding what the PV curve is for a 0.5° pixel (the current resolution of many VOD products) remains a massive scaling challenge (Binks et al. 2024), and higher‐resolution VOD data will likely have similar limitations as CWC related to signal‐crossing with nonleaf or understory characteristics. Second, for both hyperspectral CWC and microwave VOD, temporal changes in remotely sensed metrics over time may indeed indicate changing plant Ψ so long as leaf expansion and canopy development have largely ceased (which is likely the case during drought, due to relationships between turgor pressure and growth) and no turnover in pixel composition occurs (e.g., senescence of the canopy or understory). However, the slope of the Ψ relationship through time will likely be overestimated by benchtop PV curves and may require additional parameterization. Third, mixed pixel issues aside, spatial patterns in CWC will be the hardest to link to tree water status as, even if biomass could be held constant, the compounding effects of leaf phenology and changes to canopy structure dampen the Ψ–LW_mass_ relationship such that area‐based metrics (LW_area_, CW_area_, and static CWC) show weak relationships with Ψ; similar differences in phenology, architecture, and morphology across trees also make PV curves a poor proxy for spatial relationships even with in situ metrics. Finally, inconsistent spatial versus temporal patterns—even after Ψ–water content relationships stabilized late in the season—suggest that “space for time” approaches that are often assumed in both remote sensing and physiological studies should be interpreted cautiously.

## 5 Conclusions

We found that the relationship between plant water potential and water content is physiologically complex, particularly at the canopy level: covariation between processes relating to leaf growth, development, and stress response must be better understood to correctly interpret remote sensing retrievals. While diel patterns of tree hydration (stemming primarily from leaf capacitance) are likely measurable using remote sensing and can be established using laboratory‐based methods, more expansive ground‐based sampling will be necessary to understand how to best measure the spatial and longer term temporal variability of drought response. At these scales, SWC and leaf capacitance both vary and influence the stability of Ψ–water content relationships. That said, the consistent seasonal in situ Ψ–water content relationship and significant Ψ–dCWC relationship through time speak to the potential power of high spatial and temporal resolution VSWIR data. The outcomes of this research will become increasingly relevant with the recent and upcoming deployment of several spaceborne imaging spectrometers, including EMIT, EnMAP, Tananger, and NASA's SBG designated observable.

## Author Contributions

**Indra Boving:** conceptualization, data curation, formal analysis, investigation, methodology, project administration, resources, supervision, validation, visualization, writing – original draft, writing – review and editing. **Jean Allen:** conceptualization, data curation, methodology, writing – review and editing. **Philip G. Brodrick:** data curation, funding acquisition, investigation, methodology, project administration, resources, validation, writing – review and editing. **K. Dana Chadwick:** conceptualization, data curation, funding acquisition, investigation, methodology, project administration, resources, supervision, writing – review and editing. **Anna Trugman:** conceptualization, conceptualization, formal analysis, formal analysis, funding acquisition, investigation, project administration, project administration, resources, resources, supervision, supervision, writing – review and editing, writing – review and editing. **Leander D. L. Anderegg:** conceptualization, formal analysis, funding acquisition, investigation, methodology, project administration, resources, supervision, visualization, writing – original draft, writing – review and editing.

## Conflicts of Interest

The authors declare no conflicts of interest.

## Supporting information


Data S1.

## Data Availability

The data and code that support the findings of this study are openly available in Zenodo at https://doi.org/10.5281/zenodo.15110087. Reflectance data was obtained from ORNL DAAC at https://doi.org/10.3334/ORNLDAAC/2376.
