# Supplementary material for: The Unstable Relationship Between Drought Status and Leaf Water Content Complicates the Remote Sensing of Tree Drought Stress
Source: Glob Chang Biol. 2025 Apr 18;31(4):e70188. doi: 10.1111/gcb.70188 (PMC12007071; doi:10.1111/gcb.70188)
Supplement: Supplementary file 1 — Data S1. [file GCB-31-e70188-s001.docx]

**Supplementary information for:** The unstable relationship between drought status and leaf water content complicates the remote sensing of tree drought stress

**Table S1.** Experimental design and sample sizes across dates, times (predawn vs. midday), and species. Dates are those on which in-situ data were collected, within 6 days of overflight dates (shown in Fig 1). Species codes: *Quercus douglasii* = QUDO, *Quercus agrifolia* = QUAG. A total of 39 *Q. douglasii* trees were sampled, and 14 *Q. agrifolia*; not all trees were sampled at each date, but all were sampled at least four times during the growing season.

| Date | time | QUDO | QUAG | Measurements taken |
| --- | --- | --- | --- | --- |
| 3/8/22 | md | 20 | 13 | Psi, LWC, LWA, Al:As, LMA, SWC |
| 3/8/22 | pd | 13 | 5 | Psi, LWC, LWA, LMA |
| 3/15/22 | md | 9 |  | Psi, LWC, LWA, LMA |
| 3/15/22 | pd | 12 |  | Psi, LWC, LWA, LMA |
| 3/25/22 | md | 9 | 6 | Psi, LWC, LWA, Al:As, LMA |
| 3/25/22 | pd | 14 | 7 | Psi, LWC, LWA, LMA |
| 3/30/22 | pd | 19 | 8 | Psi, LWC, LWA, SWC |
| 4/4/22 | pd | 22 | 10 | Psi, LWC, LWA, LMA |
| 4/4/22 | md | 20 | 7 | Psi, LWC, LWA, LMA |
| 4/11/22 | pd | 20 |  | Psi, LWC, LWA, Al:As, LMA, SWC |
| 4/11/22 | md | 31 |  | Psi, LWC, LWA, LMA |
| 4/25/22 | md | 36 | 11 | Psi, LWC, LWA, LMA |
| 4/25/22 | pd | 36 | 13 | Psi, LWC, LWA, LMA |
| 5/9/22 | md | 37 | - | Psi, LWC, LWA, Al:As, LMA |
| 5/9/22 | pd | 23 | - | Psi, LWC, LWA, LMA |
| 5/23/22 | md | 32 | 13 | Psi, LWC, LWA, Al:As, LMA, SWC |
| 5/23/22 | pd | 39 | 14 | Psi, LWC, LWA, LMA |
| 7/19/22 | md | 36 | 13 | Psi, LWC, LWA, Al:As, LMA, SWC |
| 7/19/22 | pd | 33 | 12 | Psi, LWC, LWA, LMA |
| 8/18/22 | md | 15 | - | Psi, LWC, LWA, LMA, Al:As |
| 8/18/22 | pd | 16 | - | Psi, LWC, LWA, LMA |
| 9/15/22 | md | 35 | 14 | Psi, LWC, LWA, Al:As, LMA, SWC |
| 9/15/22 | pd | 35 | 13 | Psi, LWC, LWA, LMA |


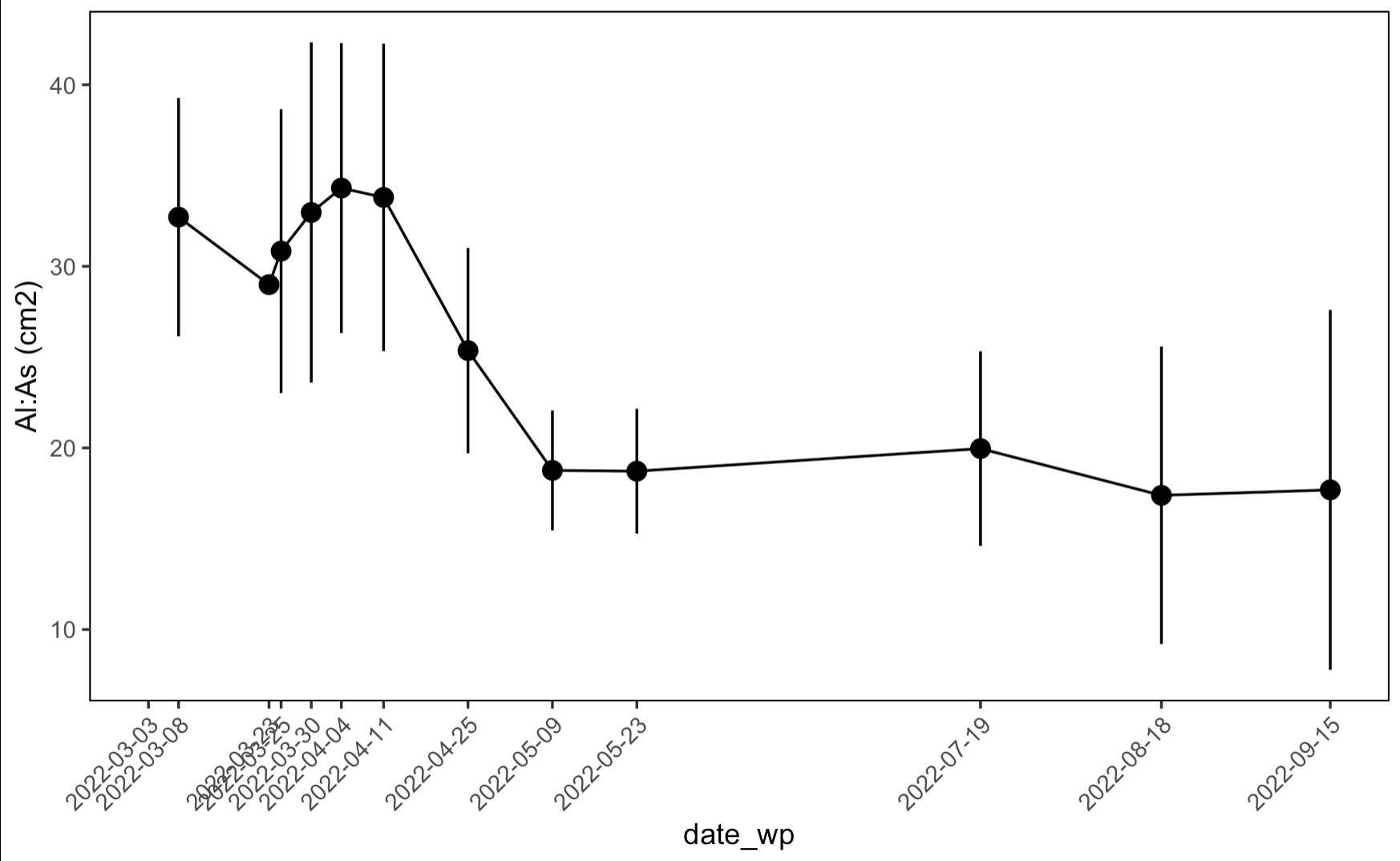


**Fig S1**. Leaf area to sapwood area (A_L_:A_S_) ratio for *Q. douglasii* over the early growing season and into the summer months.


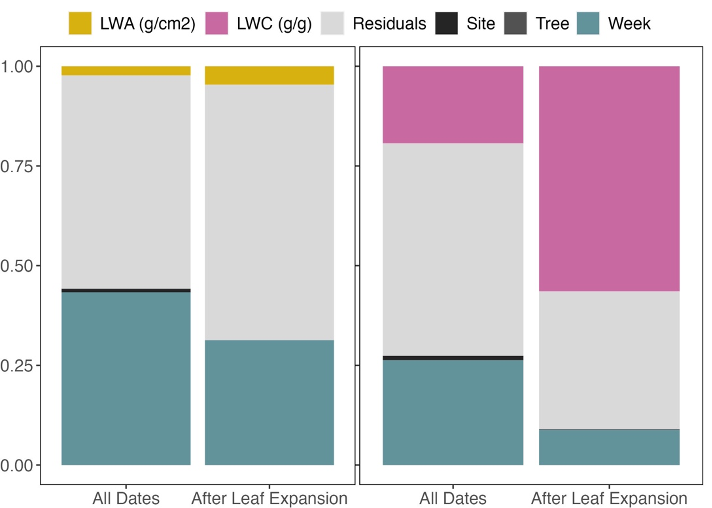


**Fig S2.** Leaf water content is related to water potential when phenology is accounted for in *Q. douglasii*. Variance decomposition of Ψ for the whole date range (March-Sept 2022) and only after leaf expansion (May - Sept 2022), for factors contributing to estimates of water potential; physical hydration metrics separated by mass-based metric (pink, LW_mass_) and area-based metric (yellow, LW_area_). Colors indicate the contribution of a given variable towards explaining variation in Ψ, with blue indicating ‘week’ variable, dark grey representing variation described by differences across trees, black representing site differences, light grey indicating residual, or unexplained, variance, and pink and yellow indicating the physical hydration metric used. Leaf expansion determined using plateaus in leaf mass per area, identified as the week of April 27th, 2022.


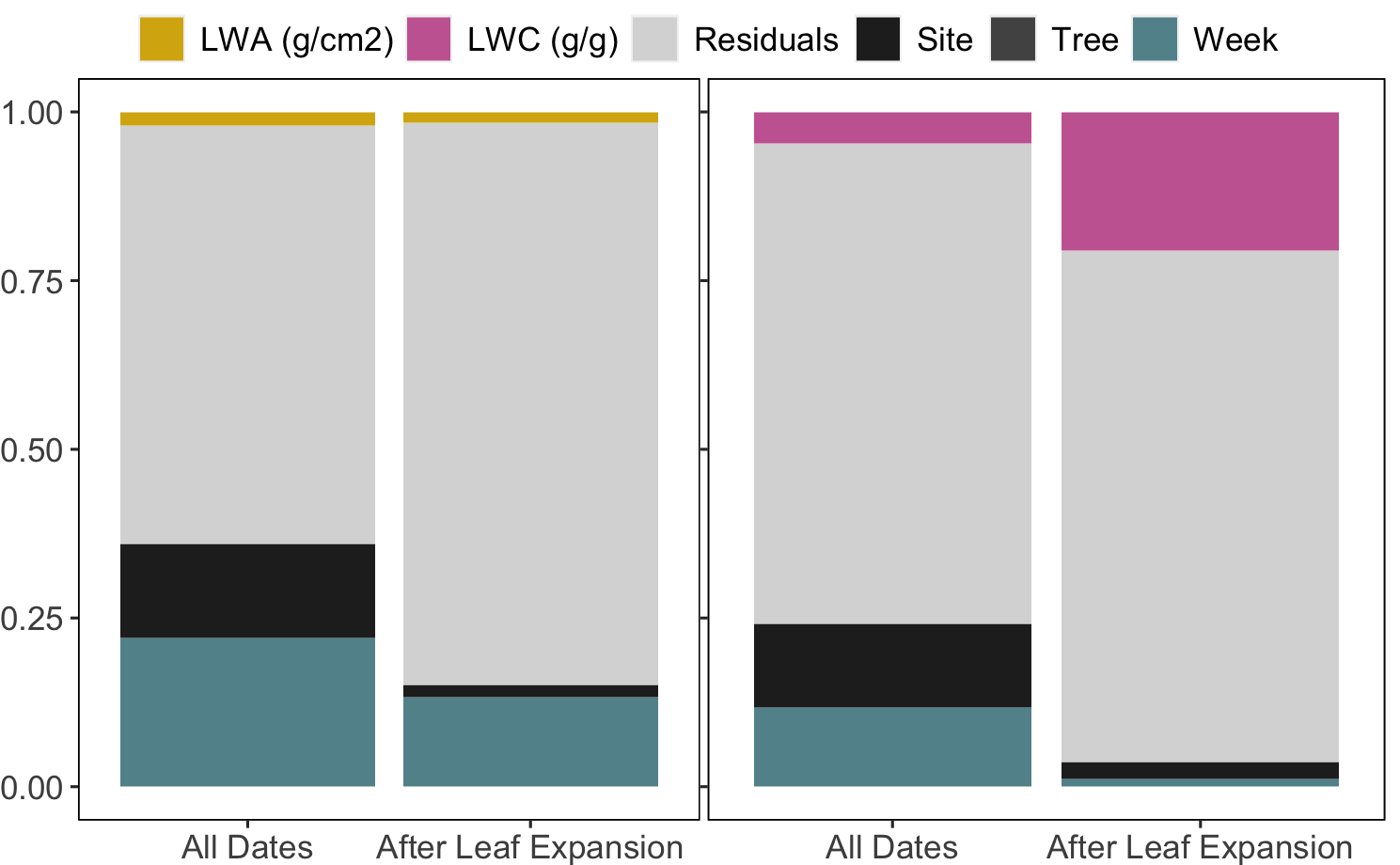


**Fig S3**. Leaf water content is related to water potential when phenology is accounted for in *Q. agrifolia*. Variance decomposition of Ψ for the whole date range (March-Sept 2022) and only after leaf expansion (May - Sept 2022), for factors contributing to estimates of water potential; physical hydration metrics separated by mass-based metric (pink, LW_mass_) and area-based metric (yellow, LW_area_). Colors indicate the contribution of a given variable towards explaining variation in Ψ, with blue indicating ‘week’ variable, dark grey representing variation described by differences across trees, black representing site differences, light grey indicating residual, or unexplained, variance, and pink and yellow indicating the physical hydration metric used. Leaf expansion determined using plateaus in leaf mass per area, identified as the week of April 27th, 2022.

**
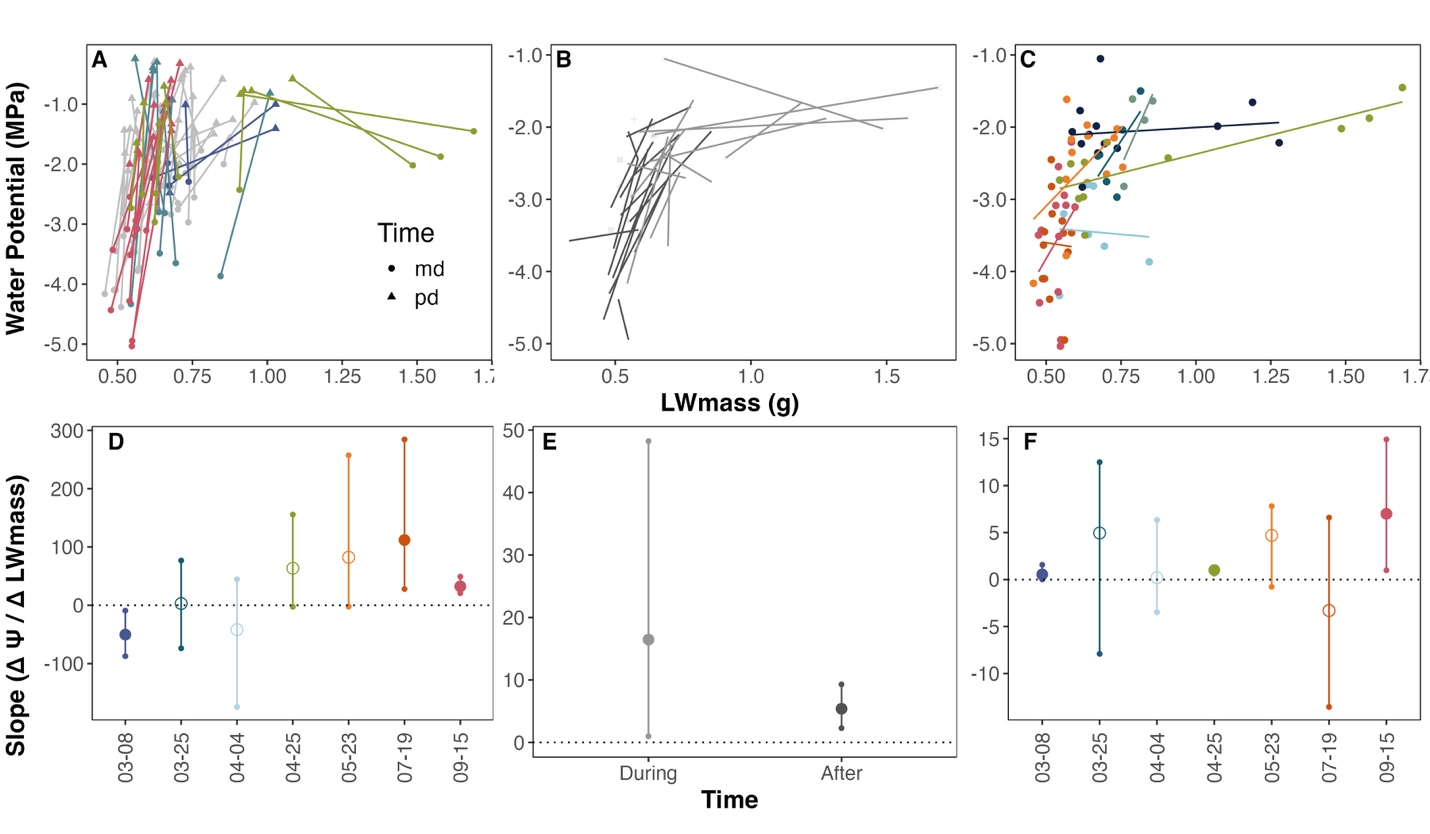
Fig S4.** The relationship between leaf water potential (Ψ) and mass-based water content (LW_mass_) varies across hydration levels, time and space for *Q. agrifolia*. A) Diel Ψ~ LW_mass_ relationship (predawn = triangles, midday = circles) with select weeks highlighted; B) Relationship between LW_mass_ and Ψ over time for each tree, separated by time periods *during* (gray) or *after* (black) leaf expansion (before or after April 27); C) Spatial variation in LW_mass_ and Ψ among trees for each sampling week; D) Mean slope +/- 95% CI across diel tree random effect; E) Mean slope +/- 95% CI across all trees for each time period. F) Slope +/- 95% CI of all trees sampled in a single week.


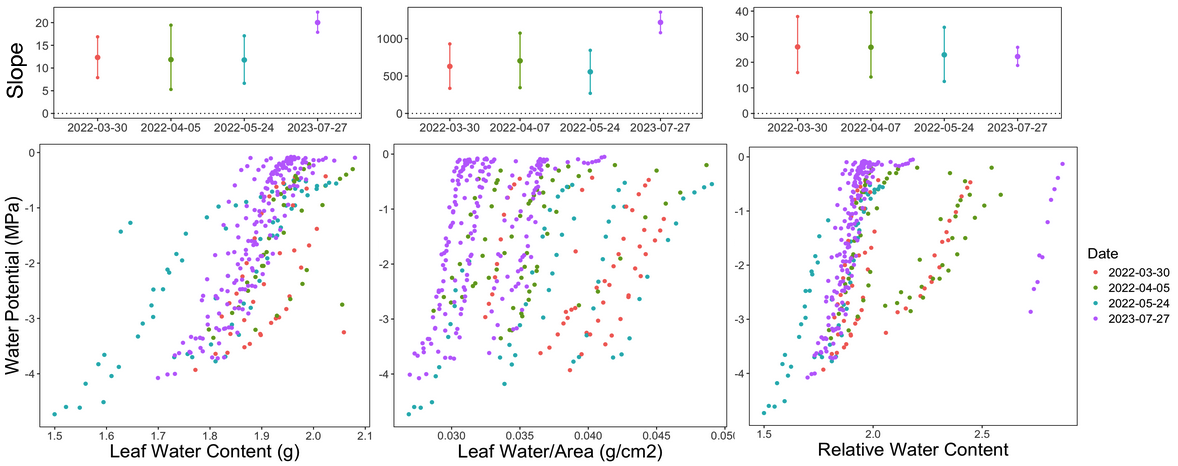


**Fig S5.** Laboratory PV curves for *Q. douglasii* with bootstrapped slopes. PV curves are plotted with LW_mass_ (bottom left), LW_area_ (bottom center), as well as the traditional relative water content (RWC; bottom right). Top row shows average slopes and 95% confidence intervals for each sampling date (colors). Sample sizes: 3/30: 6 trees, 4/07: 3 trees, 5/24: 6 trees, 7/27/2023: 13 trees.


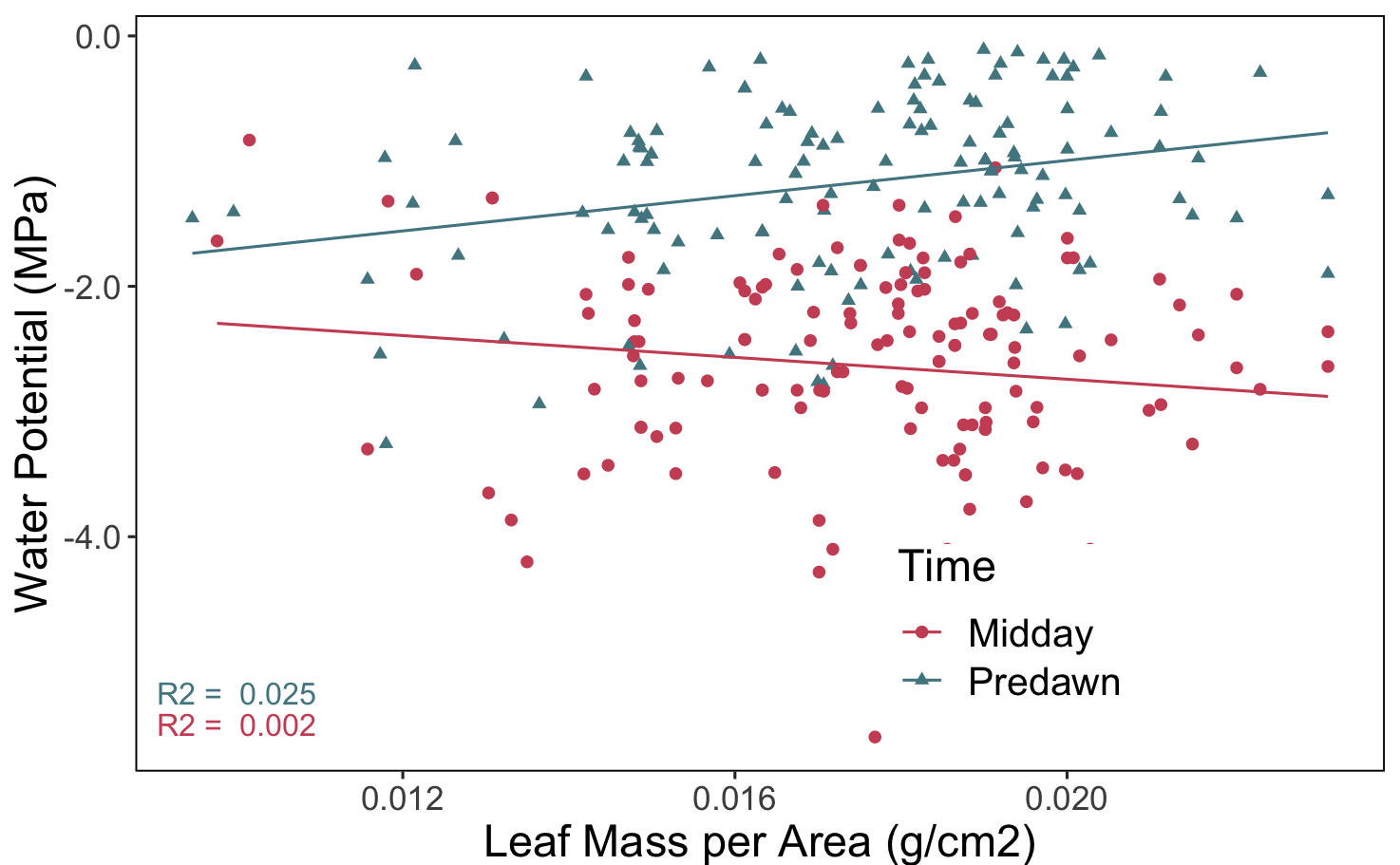


**Fig S6.** Relationship between leaf mass per area and water potential measured at predawn (blue triangles) and midday (red circles) for *Q. agrifolia* (n = 68). Marginal R2 of linear mixed-effects models with tree random intercepts shown in red or blue. LMA from samples measured on exact leaves used for water potential measurements, and from samples taken separately at a proximal date.


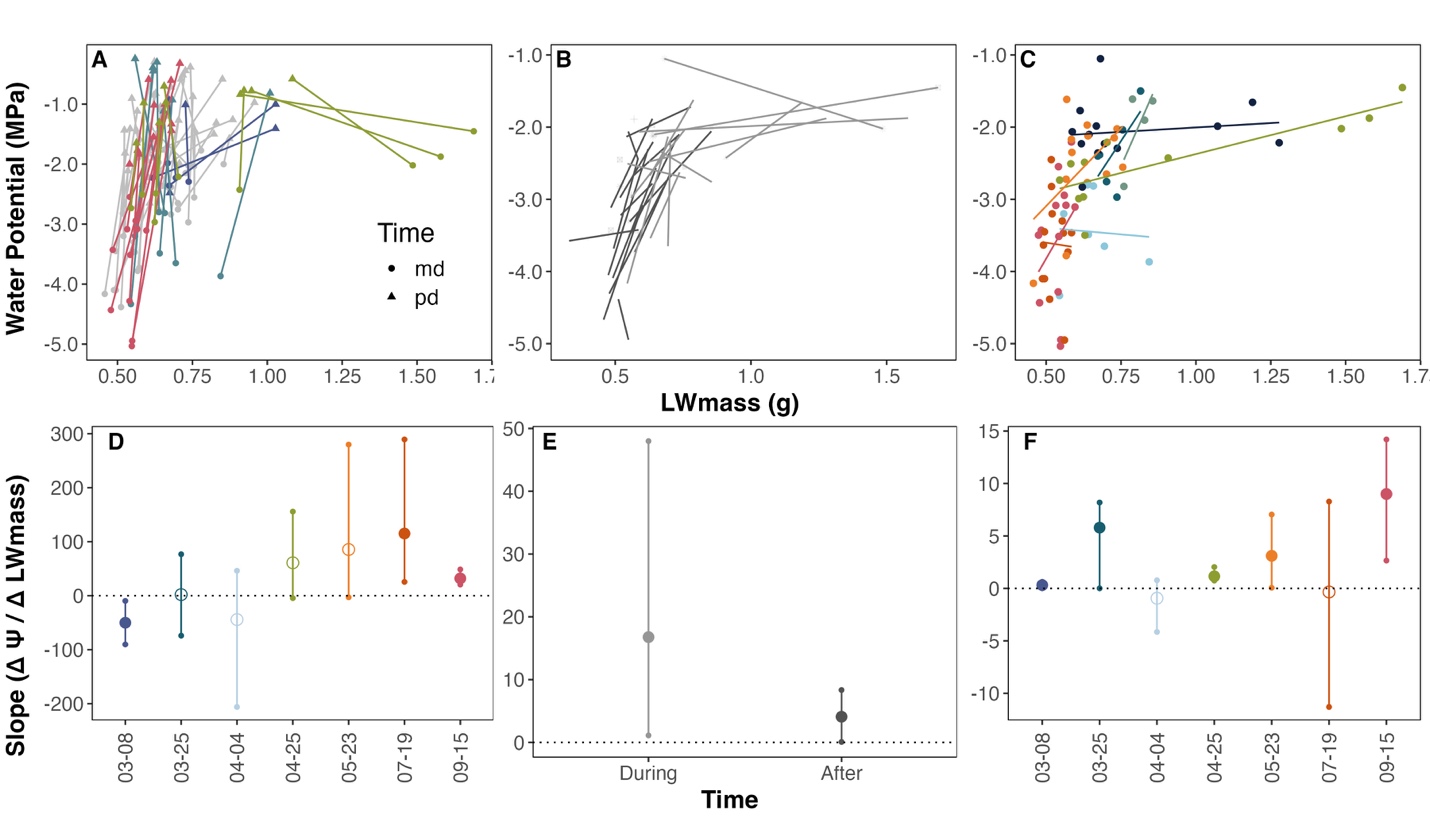


**Fig S7.** The relationship between water potential (Ψ) and area-based water content (LW_area_) varies across hydration levels, time and space for *Q. agrifolia*. A) Diel Ψ~LW_area_ relationship (predawn = triangles, midday = circles) with select weeks highlighted; B) Relationship between LW_area_ and Ψ for each tree measured over time, separated by time periods *during* (gray) or *after* (black) leaf expansion (before or after April 27); C) Spatial variation in LW_area_ and Ψ among trees for each sampling week; D) Mean slope +/- 95% CI across diel tree random effect; E) Mean slope +/- 95% CI across all trees for each time period. F) Slope +/- 95% CI for all trees sampled in a single week (spatial effect).

**Table S2.** Model comparison statistics for each mixed-effects model analysis predicting Ψ and each water content metric. Bolded model terms indicate the relationships and slopes visualized in main figures, where ‘phenology’ indicates separation of ‘during’ vs. ‘after leaf expansion’ time periods. Diel models were built on data using both predawn and midday samplings, which together show the effects of purely hydration driven signals. Marginal and conditional R2 shown for each model, with largest marginal R2 highlighted for each analysis.

| ***Species*** | ***Var.*** | ***Model*** | ***R2m*** | ***R2c*** |
| --- | --- | --- | --- | --- |
| *Q. dougliassii* | *Space*  Ψ | ~ CWC + (**CWC \|week)** + (1\|site) | 0.005 | 0.802 |
|  |  | ~ LW_mass_ + (**LW_mass_\|week)** + (1\|site) | **0.328** | **0.809** |
|  |  | ~ LW_area_ + (**LW_area_\|week)** + (1\|site) | 0.041 | 0.900 |
|  |  | ~ LMA + **(LMA\|week)** + (1\|site) | 0.208 | 0.449 |
|  |  | ~ CW_area_ + (**CWarea\|week)** + (1\|site) | 0.003 | 0.876 |
|  | *Time* Ψ | ~ CWC + **phenology** + (1\|week) + **(CWC \|tree)** | 0.257 | 0.782 |
|  |  | ~ LW_mass_ + **phenology** + (1\|week) + **(LW_mass_\|tree)** | **0.667** | **0.846** |
|  |  | ~ LW_area_ + **phenology** + (1\|week) + **(LW_area_\|tree)** | 0.411 | 0.826 |
|  |  | ~ LMA + **phenology** + (1\|week) + **(LMA\|tree)** | 0.311 | 0.579 |
|  |  | ~ CW_area_ + **phenology** + (1\|week) + **(CW_area_ \|tree)** | 0.339 | 0.874 |
|  | *Diel* Ψ | ~ LW_mass_ + **(LW_mass_\|tree)** + (LW_mass_\|week) | **0.225** | **0.614** |
|  |  | ~ LW_area_ + **(LW_area_\|tree)** + (LW_area_\|week) | 0.171 | 0.825 |
| *Q. agrifolia* | *Space* Ψ | ~ CWC + (**CWC \|week)** + (1\|site) | 0.002 | 0.574 |
|  |  | ~ LW_mass_ + (**LW_mass_\|week)** + (1\|site) | **0.078** | **0.552** |
|  |  | ~ LW_area_ + (**LW_area_\|week)** + (1\|site) | 0.000 | 0.743 |
|  |  | ~ LMA + (**LMA\|week)** + (1\|site) | 0.009 | 0.203 |
|  |  | ~ CW_area_ + (**CWarea\|week)** + (1\|site) | 0.000 | 0.906 |
|  | *Time*  Ψ | ~ CWC + **phenology** + (1\|week) + **(CWC \|tree)** | 0.047 | 0.718 |
|  |  | ~ LW_mass_ + **phenology** + (1\|week) + **(LW_mass_\|tree)** | **0.227** | **0.712** |
|  |  | ~ LW_area_ + **phenology** + (1\|week) + **(LW_area_\|tree)** | 0.106 | 0.745 |
|  |  | ~ LMA * **phenology** + (1\|week) + **(LMA\|tree)** | 0.115 | 0.218 |
|  |  | ~ CW_area_ + **phenology** + (1\|week) + **(CW_area_\|tree)** | 0.174 | 0.801 |
|  | *Diel* Ψ | ~ LW_mass_ + **(LW_mass_\|tree)** + (LW_mass_\|week) | **0.225** | **0.614** |
|  |  | ~ LW_area_ + **(LW_area_\|tree)** + (LW_area_\|week) | 0.009 | 0.367 |

**
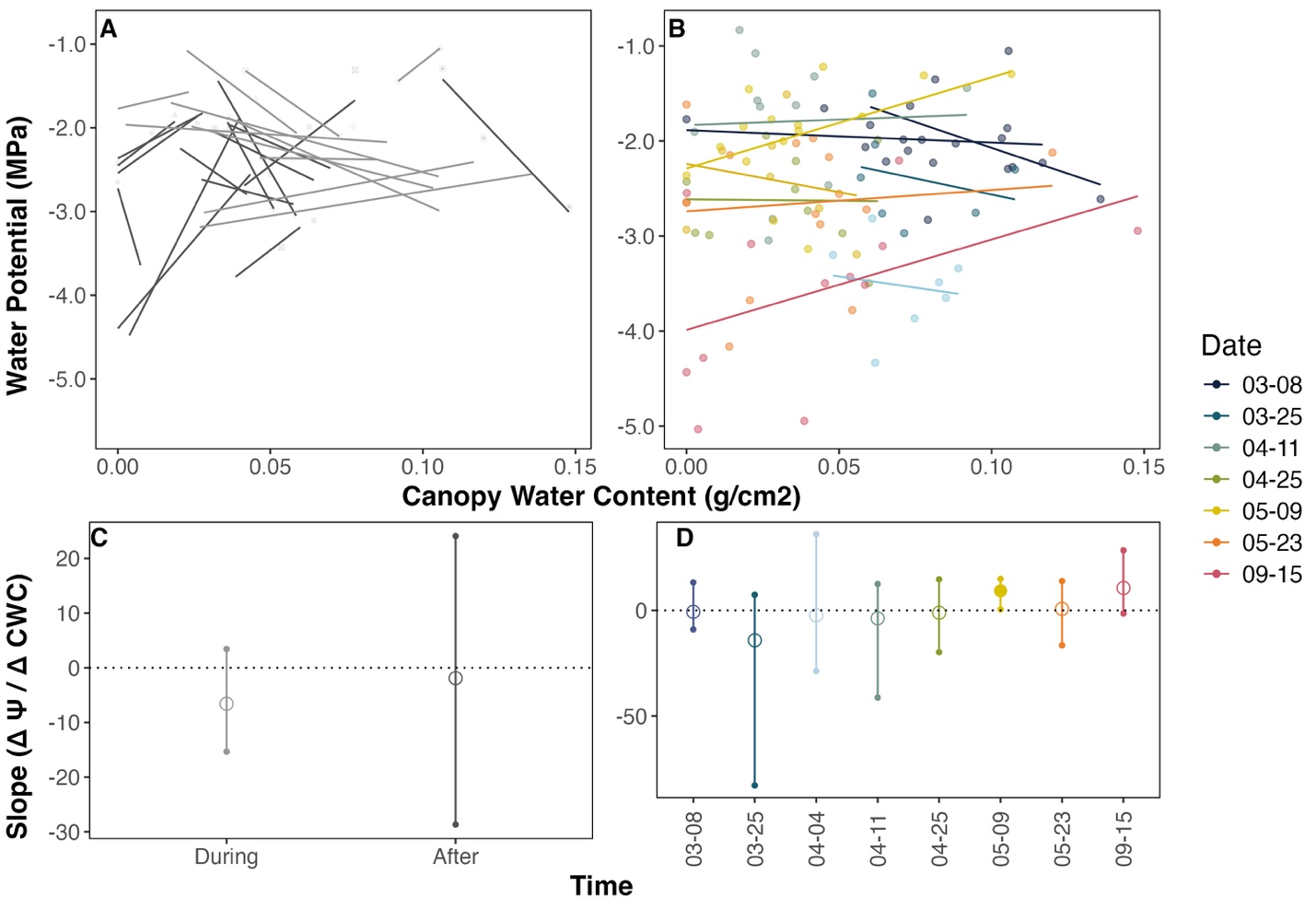
**

**Figure S8.** Leaf water potential (Ψ) as predicted by remotely sensed Canopy Water Content (CWC) in *Q. agrifolia*. A) Relationship between CWC and Ψ in individual trees before vs. after leaf expansion (April 27, 2022). B) Spatial variation in CWC and Ψ among trees for each sampling week; C) mean slope +/- bootstrapped 95% CI across all trees for each time period, separated by *before* and after *leaf* expansion; D) slope +/- 95% CI of week random effect.


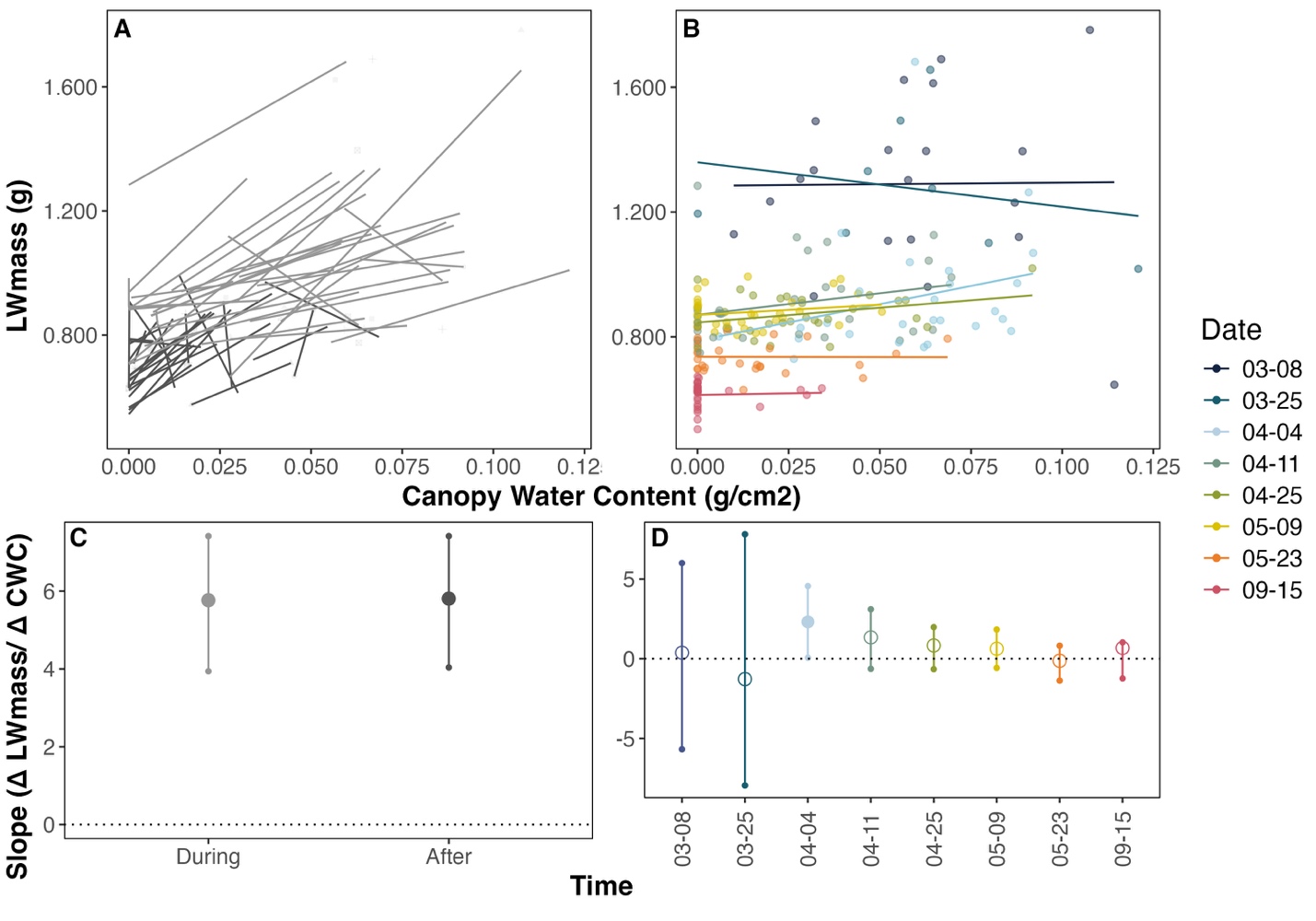


**Figure S9.** Mass-based water content (LW_mass_) as predicted by remotely sensed Canopy Water Content (CWC) in *Q. douglasii*. A) Relationship between CWC and Ψ in individual trees before vs. after leaf expansion (April 27, 2022). B) Spatial variation in CWC and Ψ among trees for each sampling week; C) mean slope +/- bootstrapped 95% CI across all trees for each time period, separated by *before* and after *leaf* expansion; D) slope +/- 95% CI of week random effect.

**
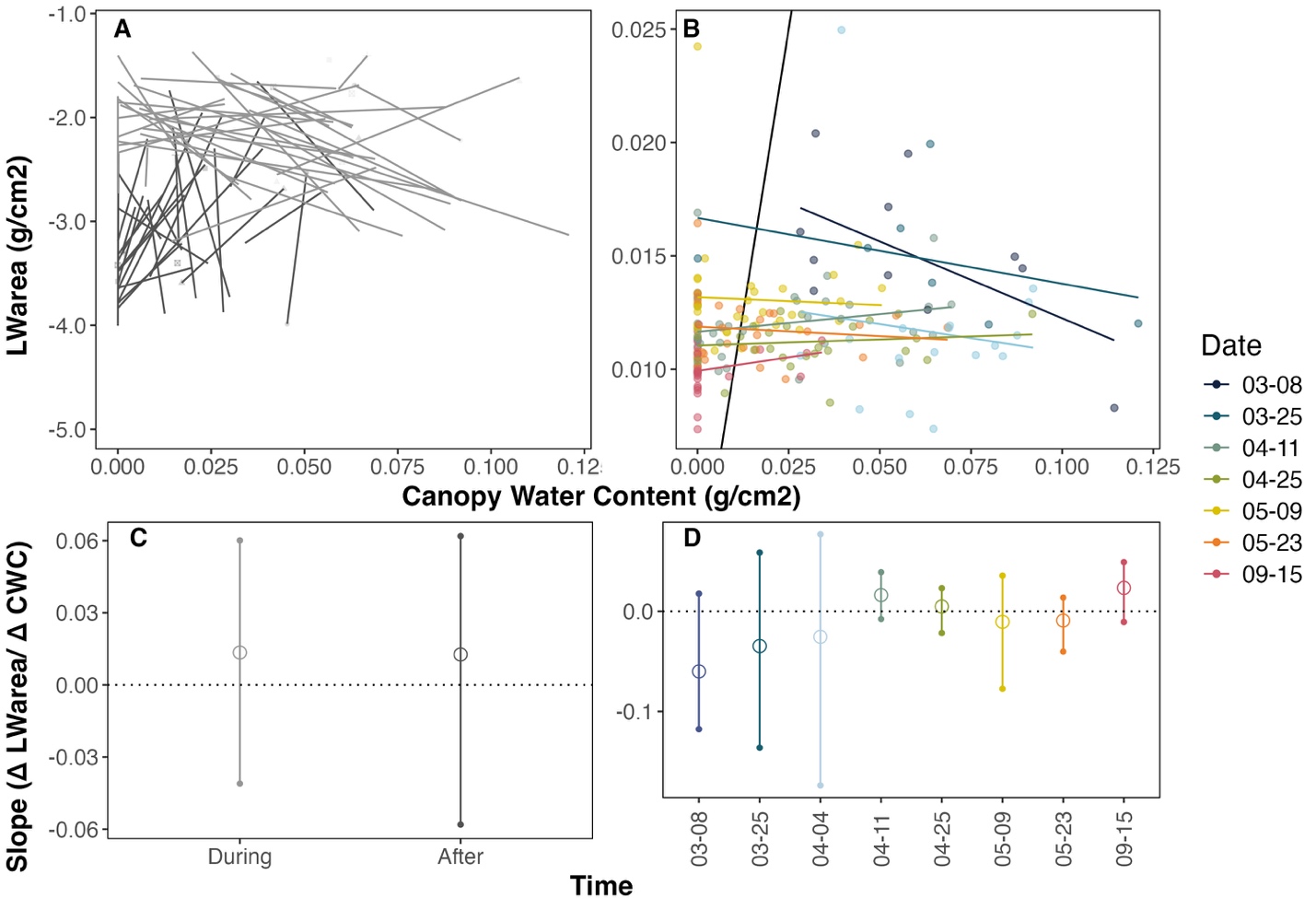
Figure S10**. Area-based water content (LW_area_) as predicted by remotely sensed Canopy Water Content (CWC) in *Q. douglasii*. A) Relationship between CWC and Ψ in individual trees before vs. after leaf expansion (April 27, 2022). B) Spatial variation in CWC and Ψ among trees for each sampling week; C) mean slope +/- bootstrapped 95% CI across all trees for each time period, separated by *before* and after *leaf* expansion; D) slope +/- 95% CI of week random effect.


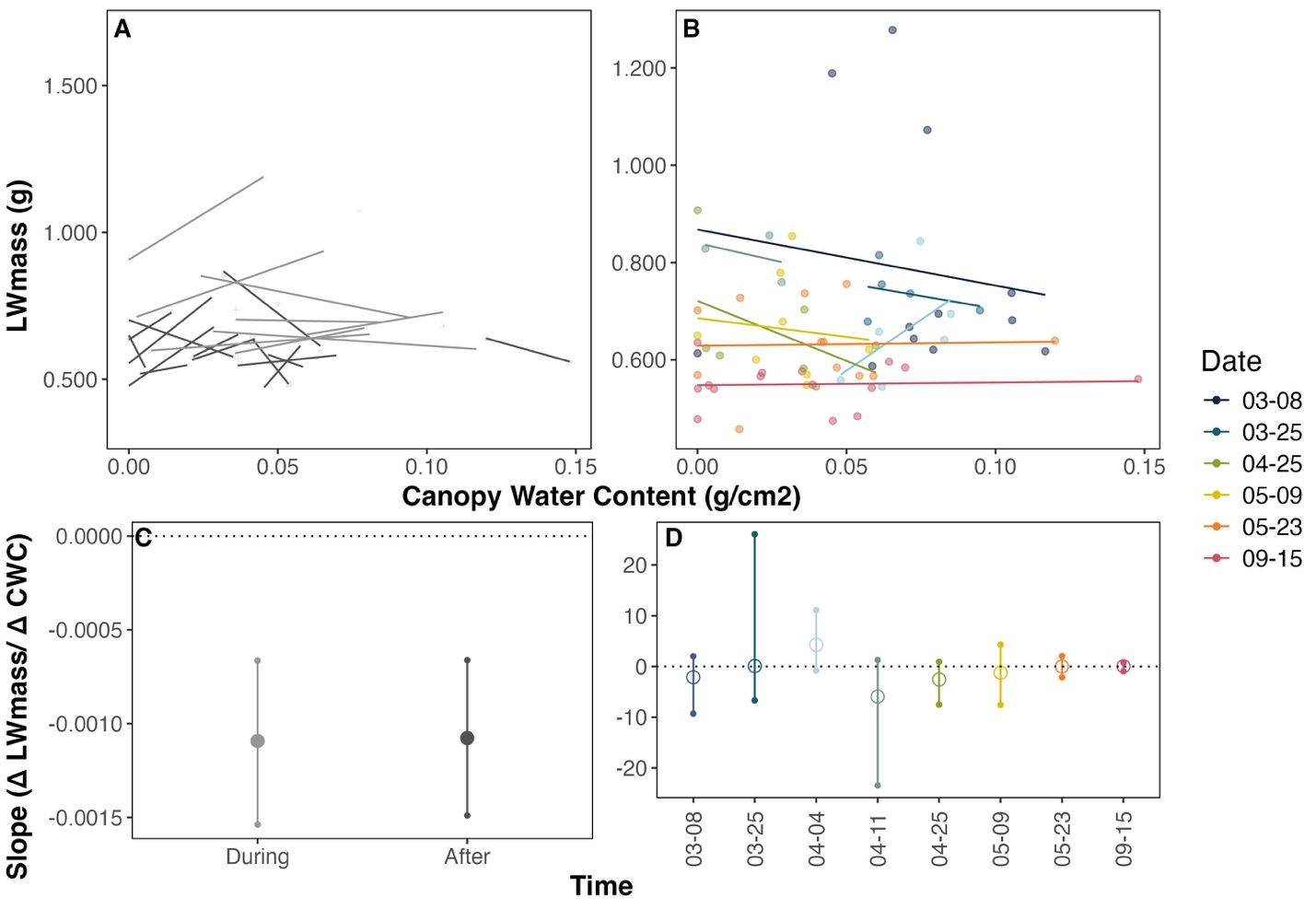


**Figure S11.** Mass-based water content (LW_area_) as predicted by remotely sensed Canopy Water Content (CWC) in *Q. agrifolia*. A) Relationship between CWC and Ψ in individual trees before vs. after leaf expansion (April 27, 2022). B) Spatial variation in CWC and Ψ among trees for each sampling week; C) mean slope +/- bootstrapped 95% CI across all trees for each time period, separated by *before* and after *leaf* expansion; D) slope +/- 95% CI of week random effect.


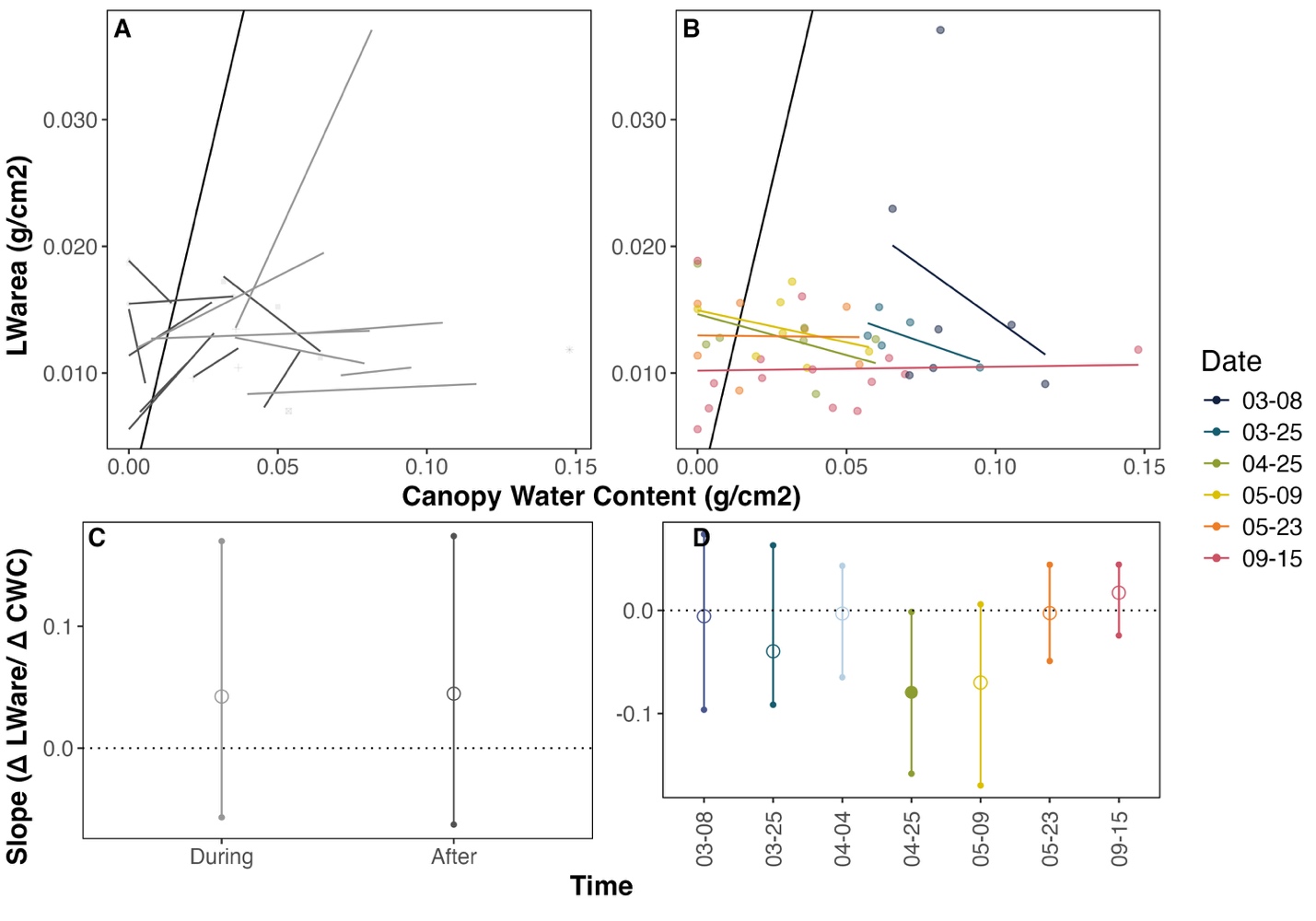


**Figure S12.** Area-based water content (LW_area_) as predicted by remotely sensed Canopy Water Content (CWC) in *Q. agrifolia*. A) Relationship between CWC and Ψ in individual trees before vs. after leaf expansion (April 27, 2022). B) Spatial variation in CWC and Ψ among trees for each sampling week; C) mean slope +/- bootstrapped 95% CI across all trees for each time period, separated by *before* and after *leaf* expansion; D) slope +/- 95% CI of week random effect.


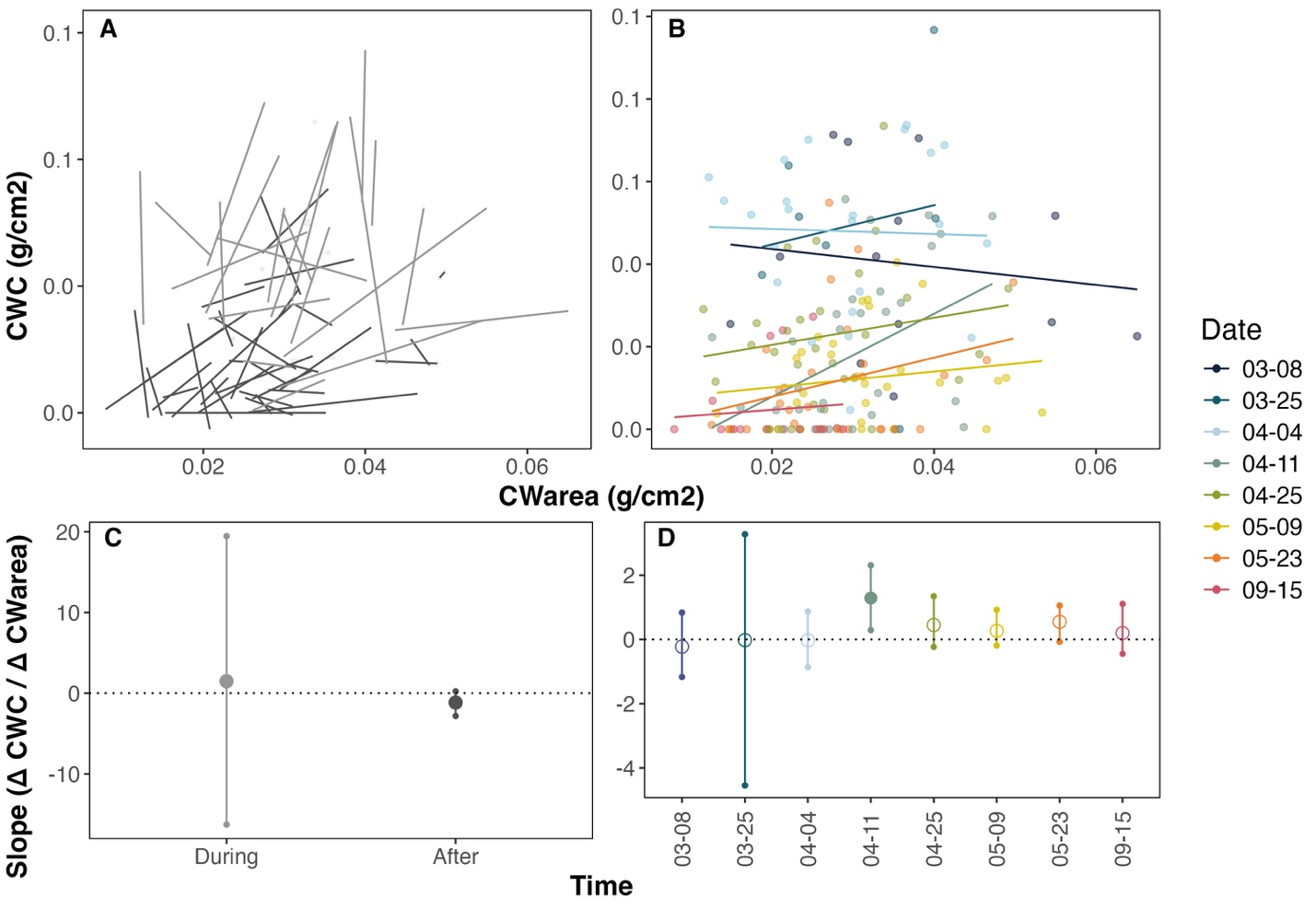


**Figure S13.** CWC from remote sensing compared to CWarea calculated from LWmass, and LAI for *Q. douglasii*. A) Relationship between CWC and CWarea in individual trees before vs. after leaf expansion (April 27, 2022). B) Spatial variation in CWC and CWarea among trees for each sampling week; C) mean slope +/- bootstrapped 95% CI across all trees for each time period, separated by *before* and after *leaf* expansion; D) slope +/- 95% CI of week random effect.

Fig S
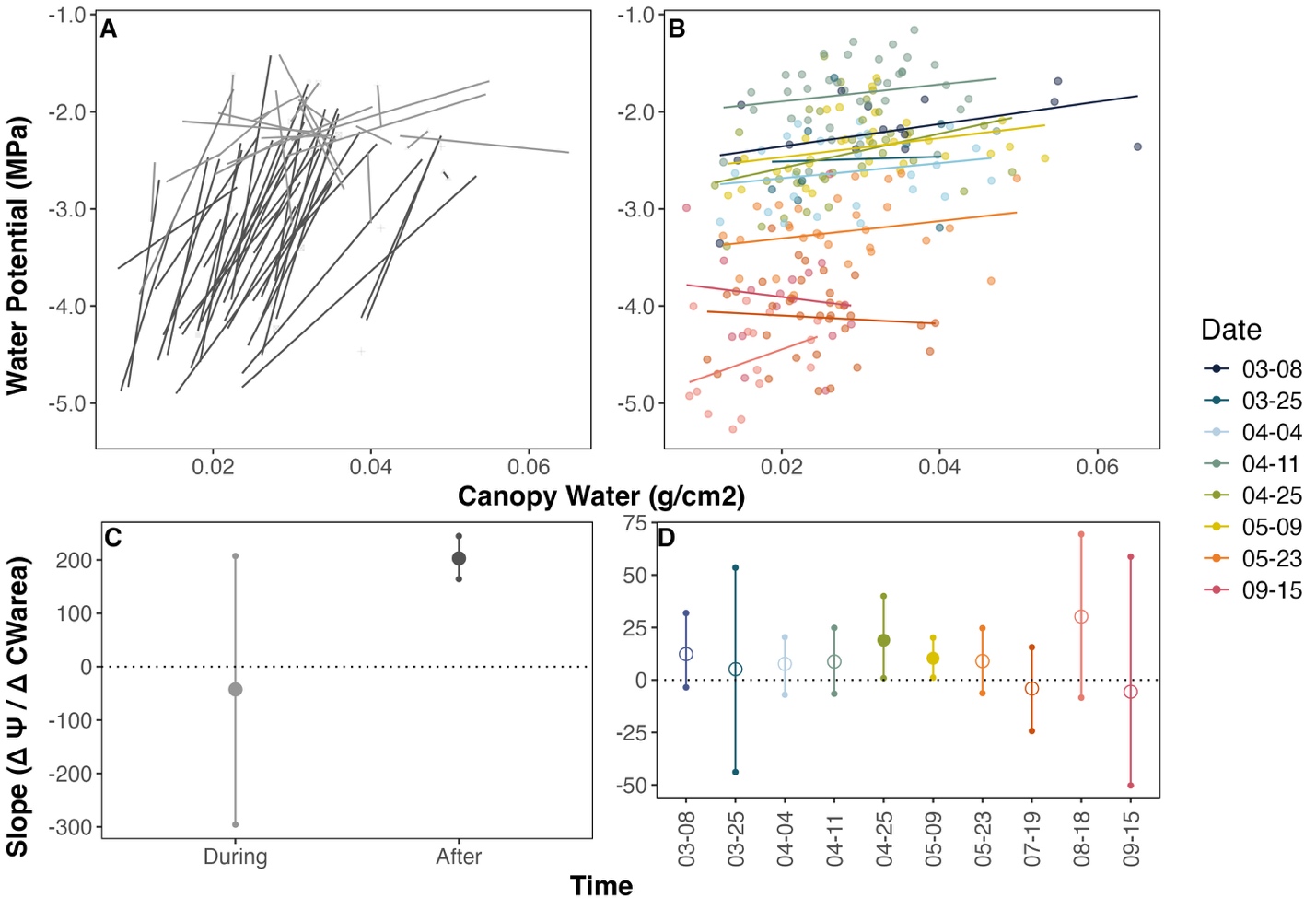
**Figure S14.** Relationship between water potential (Ψ) and CWarea calculated from LWarea, LWmass, and LAI compared to Psi for *Q. douglasii*. . A) Relationship between Ψ and CWarea in individual trees before vs. after leaf expansion (April 27, 2022). B) Spatial variation in Ψ and CWarea among trees for each sampling week; C) mean slope +/- bootstrapped 95% CI across all trees for each time period, separated by *before* and after *leaf* expansion; D) slope +/- 95% CI of week random effect.


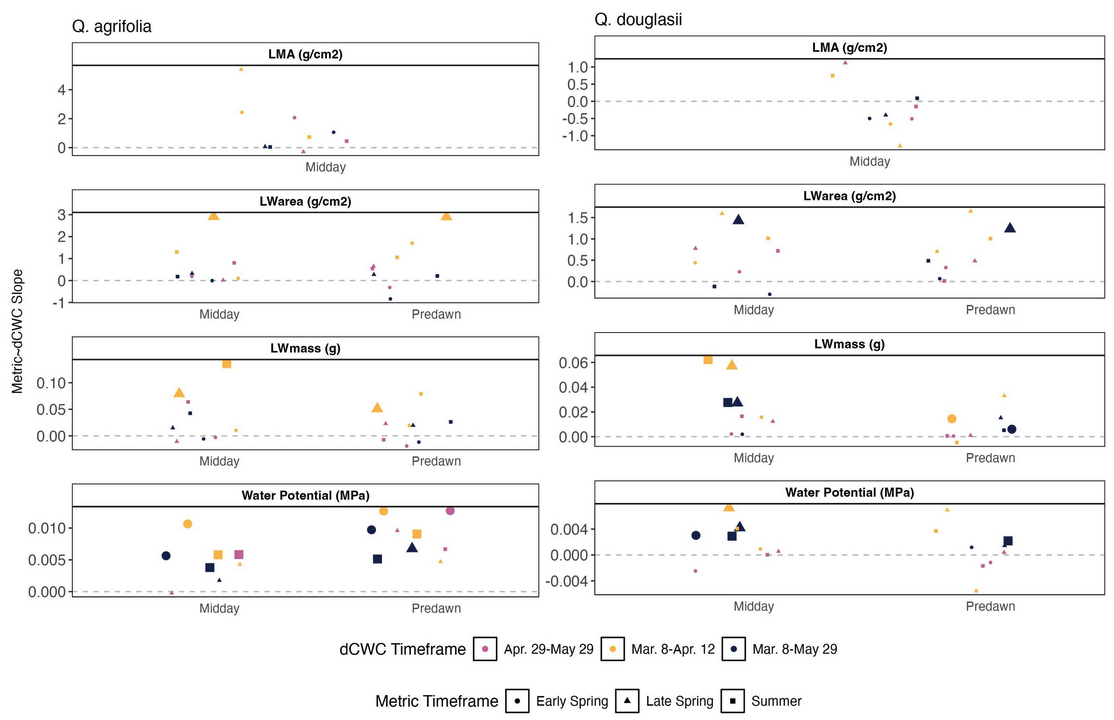


**Figure S15.** Ability for dCWC to predict in-situ metrics depends on period used to calculate dCWC and timing of in-situ sampling for two oak species: *Q. douglasii* (left panel) and *Q. agrifolia* (right panel). Points indicate slope of the relationship between dCWC and faceted in-situ metric, where large points are significant relationships in linear models (p < 0.05). Point color indicates the timeframe used to calculate dCWC, while shape indicates the period over which in-situ metrics were summarized.
